# Conservation of Distinct Genetically-Mediated Human Cortical Pattern

**DOI:** 10.1371/journal.pgen.1006143

**Published:** 2016-07-26

**Authors:** Qian Peng, Andrew Schork, Hauke Bartsch, Min-Tzu Lo, Matthew S. Panizzon, Pediatric Imaging, Neurocognition and Genetics Study, Alzheimer’s Disease Neuroimaging Initiative, Lars T. Westlye, William S. Kremen, Terry L. Jernigan, Stephanie Le Hellard, Vidar M. Steen, Thomas Espeseth, Matt Huentelman, Asta K. Håberg, Ingrid Agartz, Srdjan Djurovic, Ole A. Andreassen, Anders M. Dale, Nicholas J. Schork, Chi-Hua Chen

**Affiliations:** 1 Department of Human Biology, J. Craig Venter Institute, La Jolla, California, United States of America; 2 Department of Molecular and Cellular Neuroscience, The Scripps Research Institute, La Jolla, California, United States of America; 3 Multimodal Imaging Laboratory, Department of Radiology, University of California San Diego, La Jolla, California, United States of America; 4 Department of Cognitive Science, University of California, San Diego, La Jolla, California, United States of America; 5 Department of Psychiatry, University of California, San Diego, La Jolla, California, United States of America; 6 NORMENT, KG Jebsen Centre for Psychosis Research, Department of Psychology, University of Oslo, Oslo, Norway; 7 NORMENT, KG Jebsen Centre for Psychosis Research, Division of Mental Health and Addiction, Oslo University Hospital, Oslo, Norway; 8 VA San Diego Center of Excellence for Stress and Mental Health, La Jolla, California, United States of America; 9 Dr. E. Martens Research Group of Biological Psychiatry, Center for Medical Genetics and Molecular Medicine, Haukeland University Hospital, Bergen, Norway; 10 NORMENT, KG Jebsen Centre for Psychosis Research, Department of Clinical Science, University of Bergen, Norway; 11 Neurogenomics Division, Translational Genomics Research Institute, Phoenix, Arizona, United States of America; 12 Department of Neuroscience, Norwegian University of Science and Technology (NTNU), Norway; 13 Department of Medical Imaging, St. Olav’s University Hospital, Trondheim, Norway; 14 Norwegian Center for Mental Disorders Research (NORMENT), KG Jebsen Centre for Psychosis Research, Institute of Clinical Medicine, University of Oslo, Oslo, Norway; 15 Department of Psychiatric Research, Diakonhjemmet Hospital, Oslo, Norway; 16 Department of Medical Genetics, Oslo University Hospital, Oslo, Norway; 17 Department of Neurosciences, University of California, San Diego, La Jolla, California, United States of America; Georgia Institute of Technology, UNITED STATES

## Abstract

The many subcomponents of the human cortex are known to follow an anatomical pattern and functional relationship that appears to be highly conserved between individuals. This suggests that this pattern and the relationship among cortical regions are important for cortical function and likely shaped by genetic factors, although the degree to which genetic factors contribute to this pattern is unknown. We assessed the genetic relationships among 12 cortical surface areas using brain images and genotype information on 2,364 unrelated individuals, brain images on 466 twin pairs, and transcriptome data on 6 postmortem brains in order to determine whether a consistent and biologically meaningful pattern could be identified from these very different data sets. We find that the patterns revealed by each data set are highly consistent (p<10^−3^), and are biologically meaningful on several fronts. For example, close genetic relationships are seen in cortical regions within the same lobes and, the frontal lobe, a region showing great evolutionary expansion and functional complexity, has the most distant genetic relationship with other lobes. The frontal lobe also exhibits the most distinct expression pattern relative to the other regions, implicating a number of genes with known functions mediating immune and related processes. Our analyses reflect one of the first attempts to provide an assessment of the biological consistency of a genetic phenomenon involving the brain that leverages very different types of data, and therefore is not just statistical replication which purposefully use very similar data sets.

## Author Summary

Although functional and anatomical connections among cortical regions have been intensively explored, genetically-mediated relationships between cortical regions have not been pursued to the same degree. Identifying genetic factors that mediate these relationships among different brain subcomponents can provide insight into how the human brain is organized and functions. We have assessed the genetic relationships among cortical regions using an integrated approach that considers twin data, genotype information among a large set of unrelated individuals, and gene expression measurements from postmortem neural tissues. We looked for evidence that subsets of cortical brain regions are under common or unique genetic control. We found that the patterns of genetic relationships are highly consistent across three independent data sets and multiple lines of evidence, suggesting that the patterning of cortical surface area is strongly mediated by genetic factors and, furthermore, likely reflects underlying anatomical and possibly functional relationships among cortical brain regions.

## Introduction

The human cerebral cortex is known to be composed of functionally and anatomically specialized regions based on lesion, neurophysiological and neuroimaging studies [1]. Despite considerable individual variability in the size of cortical regions and sulcal folding patterns, the overall anatomical positioning of and functional relationship between regions are remarkably consistent across individuals, suggesting that a conserved genetically-mediated program to regulate fundamental aspects of cortical development might exist. Unfortunately, little is known about the degree to which genetic factors may contribute to this pattern. Genome-wide association studies (GWAS) have only found a small number of genetic variants with effect on human brain structures [2–8], which could be a function of size and power of those studies, but does suggest that, if a program exists, it might be attributable to the subtle influence of many genes, consistent with a polygenic basis, particularly given that certain cortical structures are highly heritable [9].

We assessed the consistency of cortical patterns likely attributable to polygenic factors in humans by comparing genetic correlations between every pair of 12 pre-defined cortical regions among 2,364 unrelated individuals, 466 twin pairs, and postmortem brain samples from 6 individuals. Each of these data sets has unique features and requires a different set of statistical modeling and data analysis techniques. As such, if the results of each reveal a consistent genetically mediated pattern among the 12 human cortical regions, it would suggest that the pattern exists. Note that most replication studies of a particular phenomenon, especially in the context of genetic association studies and GWAS, focus on replicating the actual study designs in detail and not on the biological consistency of the findings in different contexts. Thus, a complementary way of validating a particular phenomenon is to assess it using different approaches altogether, looking for independent evidence of the phenomenon of interest across all of them. A recent paper by Richiardi et al. does attempt to look for consistency of results from fMRI studies and post-mortem brain gene expression studies and is thus similar in orientation to our approach [10]. However, our studies were designed to assess genetic correlations of surface area between different brain regions that could ultimately reveal connections between those brain regions, although we did not explore ‘connectivity’ in the specialized sense discussed in the fMRI literature.

In the context of polygenic studies of patterns in human cortical brain regions, there are a number of approaches one could take, as well as important issues to consider. For example, bivariate variance components or mixed models provide an estimate of the proportion of variation in each of two phenotypes that is attributable to shared genetic factors [11]. Such analyses can be pursued via pedigree and twin studies [12–14]. Alternatively, one can leverage actual genotype information on unrelated individuals by contrasting genotypic similarity estimated over the genotyped loci with phenotypic similarity. The result would be an estimate of the genetic correlation, *r*_*g*_, which is simply the genetic covariance divided by the product of genetic standard deviations of each region [11, 15]. In more practical terms, the genetic correlation can be thought of as the degree to which the genetic determinants of two (or more) phenotypes overlap. Previously, we studied genetic correlations of cortical brain regions based on twin model [16]. In our present study, we extended the investigations to utilizing genomic data and transcriptomic data from unrelated individuals. We used standard variance component models to analyze both our sample of 466 twins (Vietnam Era Twin Study of Aging (VETSA) cohort) and a combined sample of 2,364 unrelated individuals with genome-wide genotype data from five different cohort studies (which we refer to as the “combined 5 cohort” or “C5C” sample) data. We complement these twin and genotype-based analyses with studies of the correlations among the expression levels of genes across the cortical regions in 6 postmortem brain samples [17, 18].

To pursue these analyses, we first parceled the cortex into 12 pre-defined regions. We previously used a data-driven clustering technique to identify 12 maximally genetically correlated subdivisions of the human cortical surface area based on the twins of VETSA cohort [16]. Although the boundaries of these regions are biologically meaningful, as they largely corresponding to functional specialization of the human brain, it is an open question as to the extent to which this pattern for subdividing the cortex is found in other relevant datasets.

Thus, to summarize our overall strategy we can break it into distinct steps. First, we sought to replicate and validate our genetically based cortical parcellation scheme in samples independent of those used in an original study where this parcellation scheme was identified. Second, we evaluated the genetic correlations between the cortical subdivisions based on their surface areas from genotype data on unrelated individuals and from analyses on a large sample of twins. Third, we further investigated genetic correlation profiles by leveraging transcriptomes associated with the cortical regions from 6 unrelated individuals [17, 18]. We compared the results of each of these analyses by quantifying their agreement on the patterns of correlations that emerged. This was done formally by assessing the degree of concordance between entries in the pairwise cortical region correlation matrices derived from the twin, genotype and gene expression data using the Mantel test [19]. Our overall hypothesis was that a truly conserved, genetically-mediated pattern between cortical regions, if exists, will be revealed across independent samples. We also analyzed brain transcriptome data to further explore the characteristics of the genetically-mediated pattern by finding common and unique genes expressed between any pair of cortical subcomponents.

## Results

### Applying the genetically based cortical parcellations to independent data

**Fig 1:**
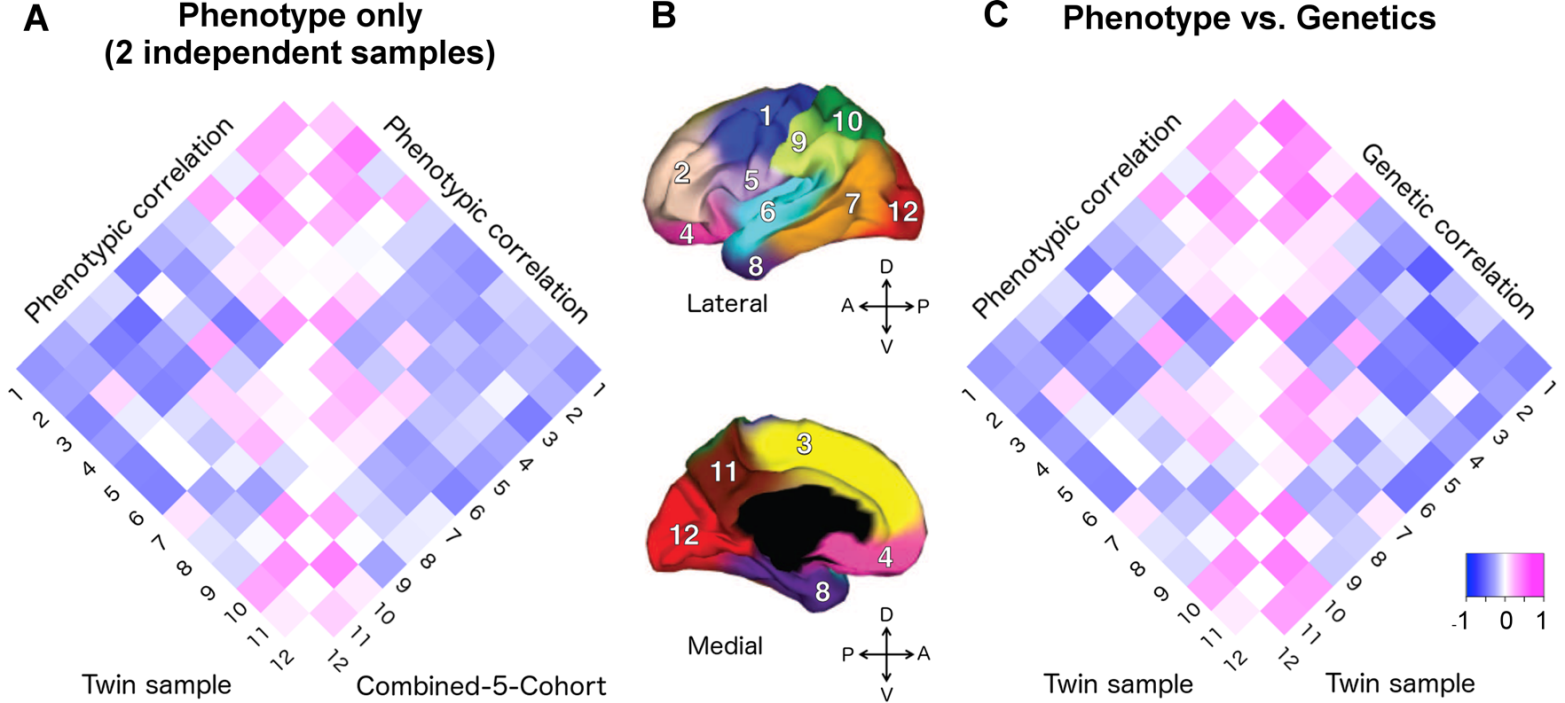
Applying the genetically based cortical parcellations to independent data. A) The phenotypic correlation matrix of VETSA twin cohort versus the phenotypic correlation matrix of combined-5-cohort (C5C). The Mantel test confirmed that the similarity between them was highly significant (*p* = 0.0001). B) Cortical brain phenotypes—surface area measures of 12 cortical regions after controlling for total surface area. The cortex was parceled into 12 genetically based regions of maximal shared genetic influence derived from the VETSA sample [16]. 1. motor & premotor; 2. dorsolateral prefrontal; 3. dorsomedial frontal; 4. orbitofrontal; 5. pars opercularis & subcentral; 6. superior temporal; 7. posterolateral temporal; 8. anteromedial temporal; 9. inferior parietal; 10. superior parietal; 11. precuneus; 12. occipital. C) The phenotypic correlation versus the genetic correlations (*r*_*g*_) matrices of VETSA. The correlation of the two matrices was also highly significant (*p* < 0.0001), suggesting high genetic contributions to the cortical patterning. Correlation coefficients are listed in Supplemental S1 Table and S2 Table.

We compared the phenotypic correlations between the VETSA twin cohort [20] and our C5C sample across 12 cortical regions. A 12x12 correlation matrix was constructed for the VETSA cohort and for the C5C sample (Supplemental S1 Table). Fig 1A depicts the two phenotypic correlation matrices as heatmaps.

We estimated the effective number of independent phenotypes from the 12 cortical regions using the eigenvalue variance metric computed from the phenotype correlation matrix. It has been shown that the total amount of correlation among a set of variables can be measured by the variance of the eigenvalues derived from the correlation matrix [21, 22], which in turn can be used to derive the effective number of independent variables (see SI method for further details). The C5C and the VETSA cohort resulted in 11.4 and 11.3 effective independent clusters, respectively. The ceiling of these numbers was 12, the number of previously genetically defined clusters, confirming the validity of the phenotypes as independent cortical factors that could lead to insight about the organization of the human brain, and its ability to stand up to independent analyses.

The phenotypic correlation matrices derived from the VETSA and C5C data had entries that were highly correlated (Fig 1A, see S1 Table for actual correlation values), with the Mantel test correlation coefficient taking on a value of 0.873 (*p*-value = 0.0001, 95% confidence interval = [0.842, 0.907]). The most highly correlated pairs of regions (correlation coefficient *r* > 0.25 in at least one data set) involved neighboring regions within the conventional lobar divisions. This pattern was consistent between the two data sets. The most anti-correlated pairs of regions, again consistent between the two data sets, were mostly between regions on the frontal lobe and regions on the other three lobes.

### Genetic correlations derived from genotype and twin analyses

As noted, the inter-cortical surface area phenotypic correlations were very similar between the VETSA and C5C sample data sets, even though the data had been collected independently. To understand what might be underlying the stability of the phenotypic correlation profile between these data sets, we investigated the underlying genetic correlations within each data set, and the similarity of the genetic correlations resulting from both data sets.

**Fig 2:**
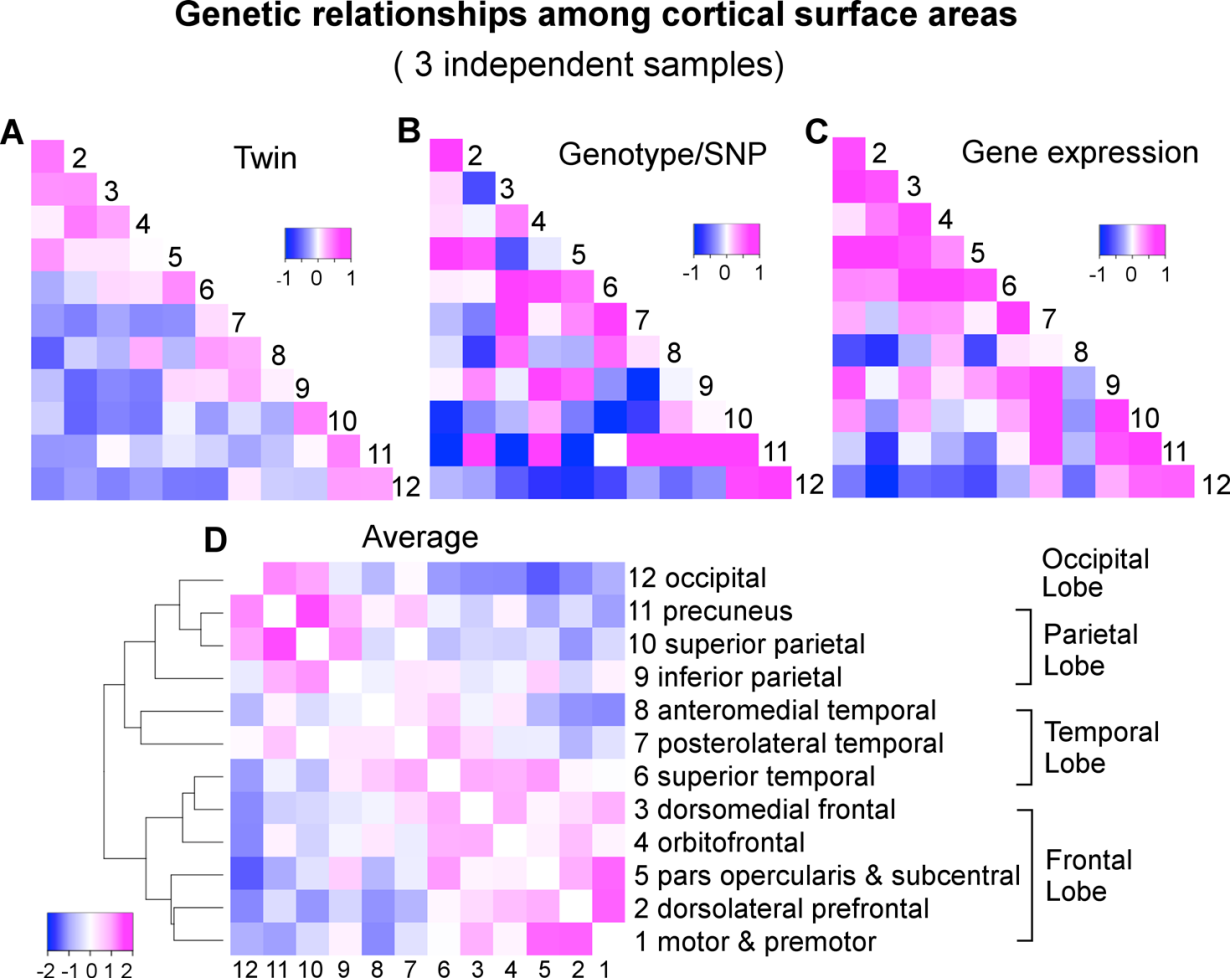
A convergent pattern of genetically mediated relationships among cortical surface areas. A) Genetic correlations (*r*_*g*_) of VETSA derived by an AE twin model. B) Genetic correlations (*r*_*g*_) of C5C derived by genotype-based GCTA-bivariate model. C) Gene expression or transcriptomic similarities of Allen Human Brain Atlas cohort based on Jaccard coefficient that are scaled to [-1,+1] such that they can be displayed on the same color scale with the correlation coefficients. Subsequent analyses were performed on the original similarity coefficients shown in S4 Table. D) Hierarchical clustering of the genetic correlations between cortical regions averaged over standardized twin *r*_*g*_ (A), genotype *r*_*g*_ (B), and gene expression similarity (C).

We applied a classical twin-based variance component model to the VETSA twin samples to derive the pairwise genetic correlations of surface area between the 12 cortical regions (S2 Table and Figs 1C and 2B). Next, we used a bivariate variance component model, as implemented in GCTA-bivariate analysis [23], to obtain estimates of the genetic correlations of the same set of phenotypes in the C5C sample data (S3 Table and Fig 2B). The elements of the cells forming the major off-diagonal triangle in Fig 2C were scaled to [-1,+1] so they would be shown on the same color scale as the other two correlation coefficient matrices in which the original values are shown. The original similarity coefficients for each matrix are listed in S2 Table, S3 Table, S4 Table, and used in all analyses. We emphasize that we assessed the consistency of the patterns of correlation between brain regions across the data sets and not necessarily the equivalence of the actual correlation strength between pairs of brain regions across the data sets.

To assess the similarity of the estimates of genetic correlations between cortical regions across the VETSA twin and C5C sample data sets, rather than filter the results at an arbitrary threshold, we employed linear regression analysis with an errors-in-variables (EIV) model [24]. The magnitude of the genetic correlation between two traits might be very different from the overall heritability of those two traits; e.g., if two traits each have low heritability, they could still have a high genetic correlation by having the same small set of genetic variants influence each of them. Estimating genetic correlations can be problematic if the genetic variance component for one of the traits is very low, however, because the estimate of that variance component will tend to be less reliable, with a large standard error; i.e., any resulting genetic correlation involving that trait and another is likely to have a large overall error and be less reliable, no matter how robust or reliable the estimate of the other trait’s genetic variance. For example, in the extreme case of the precuneus (cluster 11), its genotype-based heritability was barely 5% [9]. As a result, the estimates of its genetic correlations with all other regions had very large errors, rendering the estimates uninformative (see column 11 and row 11 of S3 Table, and row 11 of Fig 2B). We therefore weighted the estimates of the genetic correlations by their variances in the EIV model. As a comparison, we also computed Pearson’s correlation coefficient between each pair of correlation or similarity matrices under investigation, effectively ignoring any estimation errors. An overall test of the similarity of the genetic correlations across the VETSA and C5C data sets was based on the Mantel test as described in the SI Methods.

**Fig 3:**
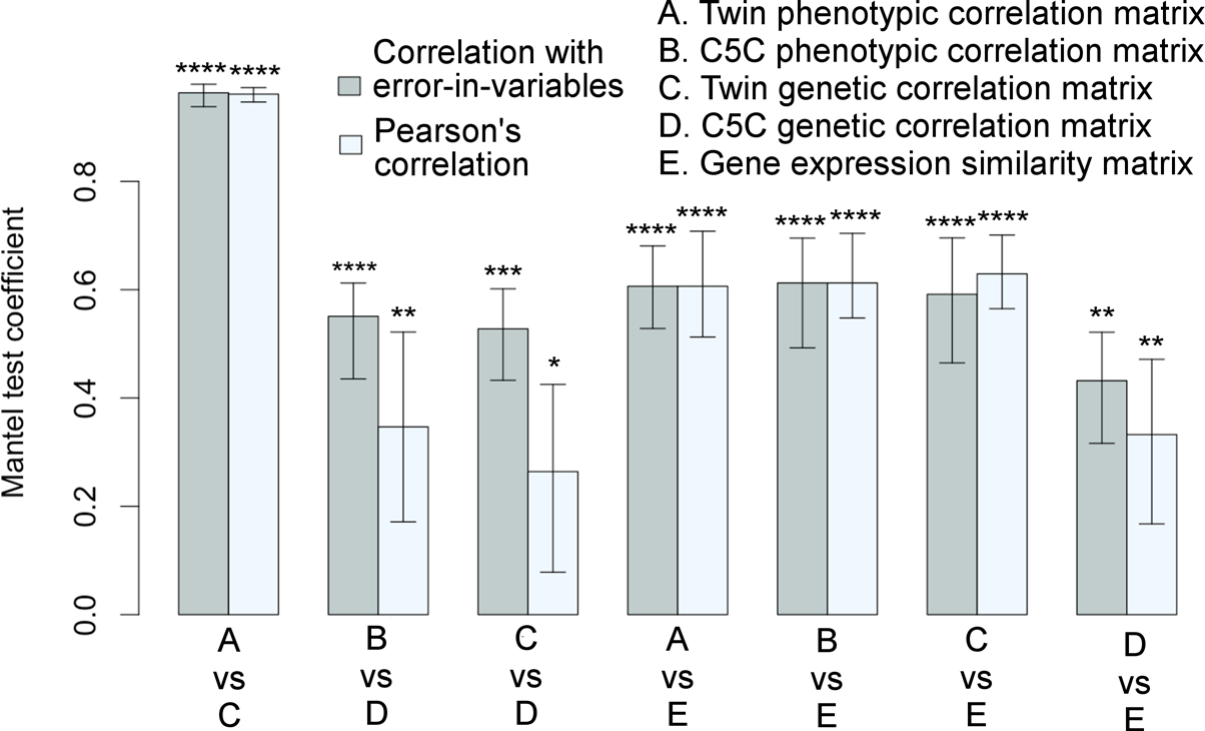
Significant associations among correlation matrices. Correlation matrices among cortical surface areas derived from a variety of measures are highly consistent with each other as quantified by the Mantel test coefficients with 95% confidence intervals. Each pair of bars represents two correlation methods used in the Mantel test: linear regression with errors-in-both-variables (correlation with EIV) in gray color on the left versus Pearson’s correlation in light-gray color on the right. Variables were standardized in regression analysis. *p≤0.05, **p≤0.01, ***p≤0.001, ****p≤0.0001. Twin refers to VETSA cohort, and twin-based method was used to derive genetic correlations. Genotype-based method was used to derive genetic correlations for ombined-5-cohort. The corresponding matrices are visualized in Figs 1 and 2. See also S1 Fig and S2 Table, S3 Table, and S4 Table.

The Mantel test results for correlation matrix comparisons are shown in Fig 3 (The actual correlation values are listed in S2 Table, S3 Table and S4 Table). The extremely high correlation between the phenotypic and genetic correlations of the VETSA twin cohort was expected (see also Fig 1C), as the cortical regions defined by genetically based parcellations were derived from the same cohort. Notably, the genetic patterns in the correlation matrices computed from the two data sets were also highly consistent. The two sample sets were not only independent, but also, as emphasized, required the use of very different analytical models and methods to estimate genetic correlations. Even when we did not use the EIV model, the correlation between the data sets was still statistically significant. The relationships between the two sets of genetic correlations obtained with each of the data sets are further detailed in Supplemental S1 Fig.

### Gene co-expression patterns derived from transcriptomic data

**Fig 4:**
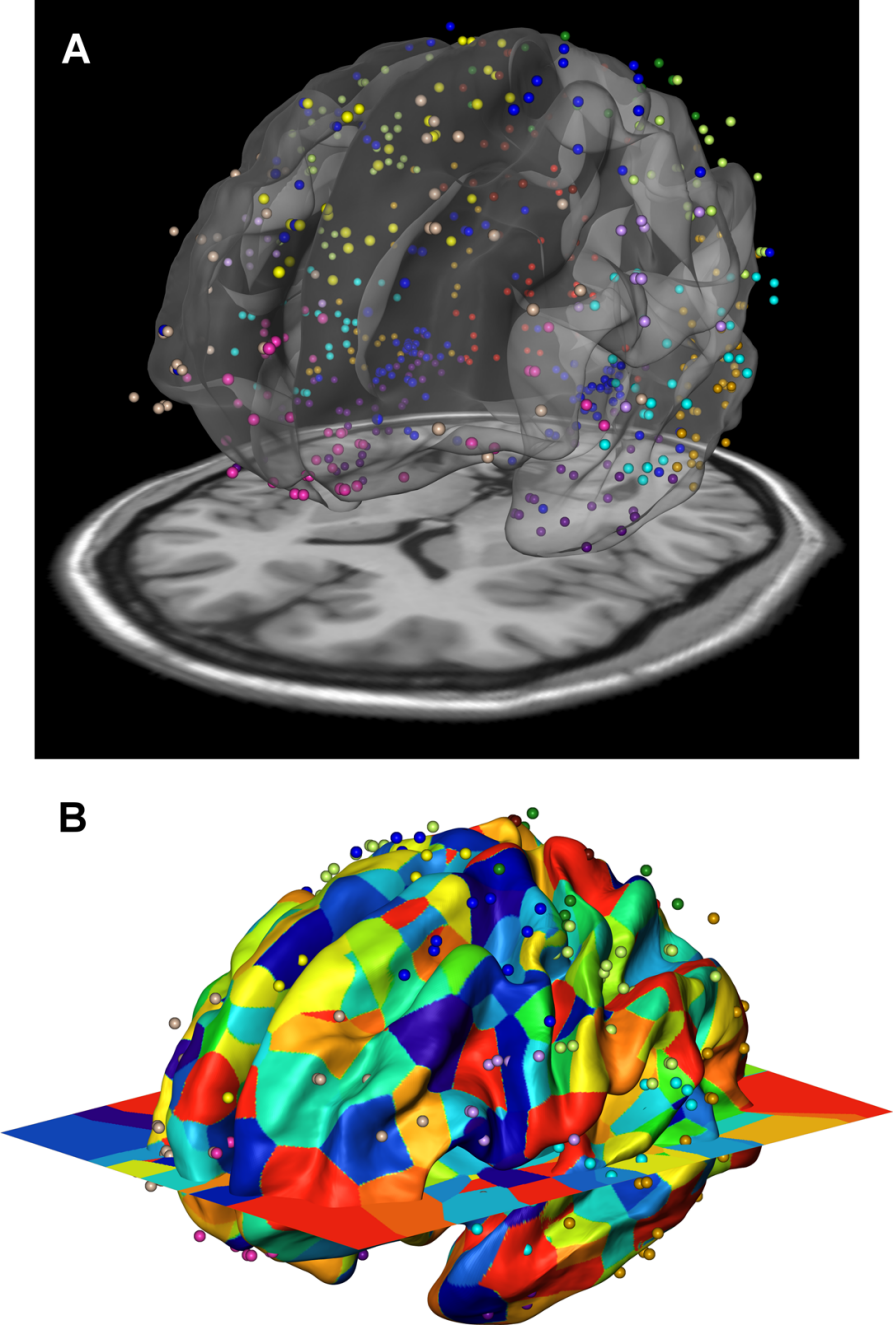
Gene expression data of the Allen Human Brain Atlas were mapped onto the 12 genetically based cortical regions in the MR space. A) Resulting volume registration between FreeSurfer surface (fsaverage) and Allen brain MNI coordinates displayed as a point cloud, with a slice of the MRI imaging at the bottom (colin27). B) After the volume registration, gene expression data points are mapped to FreeSurfer surface vertices by assigning each surface vertex the gene expression of the closest (Euclidean distance) Allen brain data point using nearest neighbor interpolation. If two vertices have the same closest Allen brain data point, they belong to the same patch and the patch id is displayed as color. Thus, the color patches illustrate the local density of data points. The color patches with similar sizes across the cortex represent an even distribution of Allen brain data points and their surface correspondences. Colors of the dots in both (A) and (B) panels represent cortical regions to which they were assigned, corresponding to the color schemes in Fig 1B.

To further investigate the phenotypic and genetic relationships of cortical surface areas, we compared the inter-regional genetic correlations with the gene co-expression profiles using a third independent data set: data from the publicly available Allen Human Brain Atlas [17, 18]. We developed a spatial mapping between the neuroanatomical subdivisions of the transcriptome data and the locations focused on in our brain imaging analysis (Fig 4). An aggregated transcriptome profile was derived for each of the 12 cortical regions we considered in the twin and genotype-based analyses. We computed inter-regional co-expression similarity profiles using the Jaccard coefficient [25], which is a unity-based normalized similarity measure. The Jaccard coefficient similarity matrix is shown in Supplemental S4 Table and the standard scores in Fig 2C.

As exhibited in Figs 1 and 2, there was generally a concordance between the transcriptome similarity matrix and the phenotypic and genetic correlation matrices. Indeed, as summarized by the Mantel test coefficients shown in Fig 3, the gene expression similarity profile was highly correlated with the phenotypic correlation profiles. These gene expression similarity profiles were also highly correlated with twin-based genetic correlations of the VETSA data, and significantly correlated with the genotype-based genetic correlations of the C5C sample data, whether the relation was obtained using linear regression based on the EIV model or a simple correlation analysis.

### Genetic correlations converge to biologically meaningful patterns

In addition to visually comparing the correlation matrices and rigorously testing the similarities between them with Mantel tests, we further examined how the genetic relationships between regions cluster those regions. We took an average over the twin genetic correlations of VETSA (Fig 2A), genotype-based genetic correlations of C5C (Fig 2B), and gene expression similarity of Allen Human Brain Atlas that were scaled to [-1,+1] (Fig 2C). We then performed a hierarchical mean linkage clustering on the averaged genetic correlations (converted to distances) between cortical regions, with the results shown in Fig 2D. The top-level cluster essentially conforms to the pattern of frontal lobe versus other lobes (temporal, parietal and occipital), with superior temporal (cluster 6) being the only exception. Within each top cluster, neighboring cortical regions are generally clustered together by their genetic correlations. We emphasize here that the genetic relations are averaged over independent datasets across different study designs with genetic correlations derived using different methodologies.

### Region-specific gene expression profiles in each lobe

**Fig 5:**
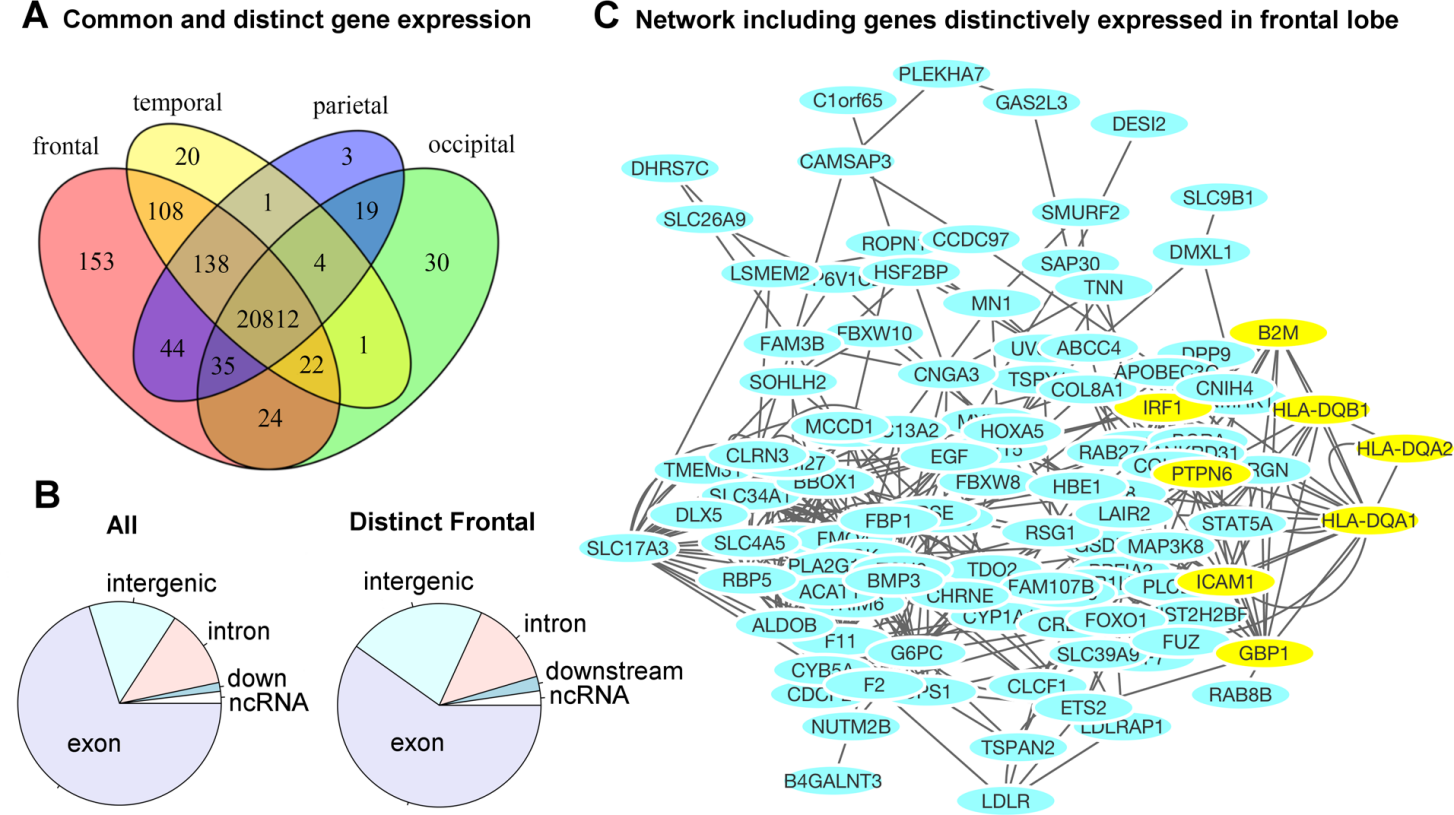
Region-specific gene expression profiles in each lobe. A) The majority of genes were ubiquitously expressed in the cortical surface areas of all four lobes of the brain. A small percentage of the genes were either distinctively expressed in one lobe or co-expressed in multiple but not all four lobes of the brain. The frontal lobe exhibits the most distinctively expressed genes. See S5 Table for the lists of genes and locations. B) The distribution of functional annotations of the transcripts distinctively expressed in the frontal lobe. “All” indicates the distribution of all transcripts included in our analysis, irrespective of their expression levels and anatomical locations. There are a higher proportion of intergenic transcripts in the frontal lobe (22% compared to 14%). C) A gene network analysis for the frontal lobe (excluding intergenic transcripts). The yellow-colored genes belong to the most significantly associated pathway: interferon-gamma-mediated signaling pathway, related to immunity (FDR = 3.2 x10^-4^). Half of the genes were originally from the transcripts distinctively expressed in the frontal lobe. See S7 Table and S8 Table for the complete list of associated pathways.

Fig 5A illustrates the number of genes distinctively expressed in the cortical regions of one lobe or co-expressed in two or more lobes of the brains. A gene is selected if it is expressed in the majority of the samples, resulting in a consensus expressed gene list for each lobe. The corresponding genes are listed in Supplemental S5 Table. The gene expression profiles of cortical regions that mapped onto the same lobe of the brain were combined to evaluate the genetic components at a gross anatomical level. A majority of the genes, 71.4%, were ubiquitously expressed in cortical surfaces of all four lobes. Approximately 2.1% of genes (602) were expressed in the cortical surfaces of at least one but not all four lobes. Fig 5B contrasts the distributions of functional annotations of all transcripts included in our analysis versus the transcripts distinctively expressed in the frontal lobe. We found a higher proportion of intergenic transcripts in the frontal lobe. A gene network analysis for the frontal lobe (excluding intergenic transcripts) is shown in Fig 5C. The genes that were used in the analysis are listed in S6 Table. The most significant pathway turned out to be the interferon-gamma-mediated signaling pathway (FDR = 3.2x10^-4^). Half of the associated genes were from the set of transcripts distinctively found in the frontal lobe. S7 Table and S8 Table list the complete list of associated functional pathways (FDR < 0.1) and the genes in the network.

## Discussion

Our integrated analyses resulted in three main findings: (1) We observed consistency of a genetically-based cortical parcellation scheme among our twin sample and C5C sample of unrelated individuals. The effective number of independent phenotypes from the 12 cortical regions was estimated to be between 11 and 12 for both sample sets, suggesting that the parcellation did identify phenotypically and genetically distinct cortical subdivisions within the spatial resolution of our brain images. (2) Genetic correlations of surface area among cortical regions estimated from twin modeling were highly consistent with those estimated from genome-wide genetic markers using an independent sample of 2,364 unrelated individuals. Similarly, the gene co-expression pattern among cortical regions in six postmortem brains was also highly consistent with genetic correlations among the surface areas of the cortical regions estimated from twin or genotype-based analysis. (3) We found that the majority of genes (71.4%) were ubiquitously expressed in the cortex, whereas the minority of genes showed region-specific expression patterns. The frontal lobe exhibited the highest number of distinctively expressed genes whose level of expression was not as pronounced in the other brain regions. These genes included some immune related genes, and a larger proportion of expressed intergenic transcripts, which we consider in more detail below.

### The consistent pattern may conform to a genetically determined prototypical pattern

How different brain structures are genetically related to each other is still something of a mystery. Here we explored the genetic relationships between cortical brain structures, asking if there is any evidence that subsets of cortical brain regions are under common or unique genetic control, and how individual cortical regions are organized genetically. To address this question, we examined evidence for pleiotropy on a whole genome basis (i.e., evidence for genetic correlations) among various cortical structures in the human brain. The genetic correlation patterns we found are highly consistent across samples (i.e., genetic correlation matrices between samples are highly correlated). For the genetic patterning to converge from different sample sets across different study designs using different analysis methods, several conditions intrinsic to all the data sets have to be met, including: (1) The boundaries of genetic cortical regions must be generally aligned; (2) the relative positions of genetic cortical regions must be preserved; (3) all pairwise genetic correlations between genetic cortical regions are similar; and (4) consistent genetic relationship estimates exist in genotype polymorphisms, genetic information based on twin data and gene expression data, regardless of data type, ethnicity, gender and age disparities across samples, because we have controlled for all these factors. Our first main finding was to test the requirements (1) and (2), and the second main finding was for the requirements (3) and (4). It is not trivial to meet all these requirements and derive convergent results from different computational models (see ‘Statistical genetic considerations’ below). We argue that this robust consistency may be driven by a genetically determined prototypical pattern or canonical cortical “blueprint” in the human brain [26].

The highly conserved genetic correlation patterns across individuals suggest that, despite substantial structural and functional variability among individuals, the rudimentary genetic patterning of the human cortex in terms of these 12 large cortical regions is fundamentally similar. Our findings are consistent with the notion of the “protomap” hypothesis, which states that regional layout of the cortex is established at early stages of development by intrinsic genetic mechanisms. The cortex is initially patterned by gradients of signaling molecules and transcription factors within cortical progenitors [27–31]. These genetic gradients exhibit spatial signatures, such as following the anterior-posterior axis, which confer positional information for initial formation of cortical areas [32, 33] and the orderly relationship of genetic effects between regions is required for proper cortical area size. These observations may explain the highly stable genetic relationships between cortical regions observed in our study.

### Genetic patterning recapitulates spatial topography

A notable feature of cortical genetic patterning is that the spatial patterns of gene expression recapitulate the spatial topography of the cortex [17]. We found strong genetic correlations among neighboring cortical regions. These proximity relationships could mirror lineage relationships of cortical neurons generated from proximal parts of the developing cortex under common influences of genetic gradients. Consistent with previous findings [16, 34], the surface area similarities between regions within the same lobe were in general higher than those between lobes, though not without a few exceptions. One such exception was the higher cross-lobe correlations in all measures between the pars opercularis and subcentral region and superior temporal region corresponding to the area associated with human language [35]. Another exception was the anteromedial temporal region. This region’s (cluster 8) expression profile stood out as being less similar to those of all other regions, which was in slight contrast to its phenotypic and genetic relationship with other regions. It is known to be involved in memory as well as more primal emotions such as fear and disgust [36]. This region also had one of the highest contributions to heritability by more conserved genomic regions [9]. The occipital cortex’s expression profile was less similar to those of other regions, but this was consistent with its phenotypic and genetic relations with those regions. Although our observed correlation pattern is not simply contributed by spatial proximity per se, exploring the spatial distributions of the genes could potentially reveal further insight into how the brain is constructed genetically.

### The frontal lobe exhibits the most distinctively expressed genes

We examined the number of genes distinctively expressed in each of the four lobes. A majority of the genes, 71.4%, were ubiquitously expressed in cortical surfaces of all four lobes. Approximately 2.1% of, or 602, genes were expressed in the cortical surfaces of at least one but not all four lobes, suggesting that subtle differences in gene expression profile in terms of spatial locations in the brain may have significant consequences for cortical functional divergence. The frontal lobe, a region showing great evolutionary expansion, showed the highest number of distinctively expressed genes. This finding was consistent with the observed negative genetic correlations between the frontal regions and the regions of the other lobes in our genotype-based and twin-based analyses (Fig 2A and 2B). The negative correlations were also observed in the coexpression matrix (Fig 2C). Note that without scaling, the similarity or correlation coefficients were in the positive scale from 0 to 1, and frontal and posterior regions were still on the opposite ends in the positive spectrum. This finding suggests the presence of polarized genetic effects on the cortical surface along the anterior-posterior axis. This lobar-based result is not contradictory to the previous analysis of the 12 regions that none of the individual frontal subdivisions showed the most distinctive genetic profile, because the frontal subdivisions are highly correlated with one another.

These selectively expressed genes in the frontal lobe are associated with immunity, cell cycle regulation and transport. The most significantly associated pathways are related to interferon-gamma, which is critical for immune response. A recent study has found that certain psychiatric disorders have involvement of immune-related gene loci [37]. The genes that appeared to be expressed to a greater degree in the different brains and contribute to the consistency of the patterns in genetic correlations between the brain regions are of interest in their own right, and should be explored for their role in disease susceptibility especially various neuropsychiatric conditions, if there are known eQTLs that affect their expression, and their association with traits via association studies, etc.

The frontal lobe also has a larger proportion of the expressed intergenic transcripts compared to the distribution of all the analyzed transcripts, suggesting stronger regulatory involvements. Intergenic transcripts have been suggested to contribute to functional differences between humans and chimpanzees in an evolutionary comparison study [38].

### Statistical genetic considerations

The genotype-based approach implemented in the GCTA program presumably captures the additive genetic effect contributed by all common single nucleotide polymorphisms (SNPs), but may not capture all the factors contributing to the heritability of a trait or phenotype (e.g., heritable components attributable to rare variants or structural variants in the genome), which are reflected in twin heritability. Hence, there is usually a difference between the overall heritability of a trait estimated from twin or pedigree data and that estimated from genotype data. Genetic correlations between two traits obtained by the twin and genotype-based analyses are likely to exhibit similar differences. Furthermore, although dominance effects at multiple loci and higher-order epistatic interactions were not explicitly considered, the consistency of the correlations across the data sets suggests that the exclusion of dominance and epistatic effects did not confound our analyses in substantive ways.

Subjects making up the unrelated individuals in the C5C sample were limited to those with European ancestry to avoid stratification and genetic background effects. The analysis method for the classic twin design and for computing gene co-expression profiles were not susceptible to mixture of genetic ancestry; therefore, those analyses included Caucasians, African Americans and Hispanics. In addition to ancestry heterogeneity, our samples contained differences in gender and age. The twin sample only had male twins between 50–59 years of age; the C5C sample had both genders between 3–90 years of age; and the postmortem brain samples also had both genders between ages 24–57. We have adjusted age and gender in the analyses. The observed consistent genetic relationships were still evident and not affected by demographic heterogeneity.

While our genetic analysis of MRI data focused on genetic correlations on one particular aspect of cortical morphology (cortical surface area), and thus implicates only those genetic elements related to cortical surface area, the transcriptome analysis we pursued examined transcriptional variation across the cortex based on all genes. Therefore, although we found consistent patterns between our genetic and transcriptome analyses at the gross anatomical level, some level of discrepancy was expected.

### Conclusions

We found a consistent pattern of genetically-mediated relationships among cortical brain regions across different data sets and different analytical techniques. These cortical brain regions are genetically defined and largely correspond to known functional specialized regions. Thus, our results suggest that the overall cortical patterning, as reflected in the relationships among cortical regions, is shaped by genetic factors and, further, that this conserved spatial pattern may be important to organize functional modules of the cortex. This robust and consistent configuration might originate from a common evolutionary and developmental pattern of cortical regionalization. Although we know that several transcription factors are key players in intrinsic genetic mechanisms of cortical regionalization, especially based on animal data, there is a large knowledge gap regarding our understanding of polygenic contribution by common genetic polymorphisms to human cortical regions. Our work sheds light on the genetically-mediated organization of cortical regionalization. Identifying the specific variants underlying the likely polygenic pleiotropic effects we observed, however, will require further, likely very large-scale, studies.

## Materials and Methods

### Ethics statement

UCSD IRB approved this study as part of Project #131068X: "The above-referenced project was reviewed and approved by one of this institution's Institutional Review Boards in accordance with the requirements of the Code of Federal Regulations on the Protection of Human Subjects (45 CFR 46 and 21 CFR 50 and 56), including its relevant Subparts." Each study was approved by the local Institutional Review Board: South East Norway (TOP and NCNG) and Mid Norway (HUNT) Regional Ethical Committee (HUNT), and UC San Diego (PING and VETSA).

### Participants

A combined sample of five sub-study cohorts (C5C) is made of 605 subjects from the Thematically Organized Psychosis (TOP) study, 842 Health Study of Nord-Trøndelag (HUNT) subjects, 325 Norwegian Cognitive Neuro-Genetics (NCNG) subjects, 726 Alzheimer’s Disease Neuroimaging Initiative (ADNI) subjects, and 1198 Pediatric Imaging Neurocognition and Genetics (PING) subjects. The samples for the twin analysis was part of the Vietnam Era Twin Study of Aging (VETSA) study [20]. There were 466 participants, of which 99 pairs were dizygotic twins and 134 pairs monozygotic twins. The sample is representative of U.S. middle-aged men in their demographic and health characteristics.

Each study was approved by the local Institutional Review Board (IRB): South East Norway (TOP and NCHG) and Mid Norway (HUNT) Regional Ethical Committee (HUNT), and UC San Diego (PING and VETSA). The current study was approved by the IRB of UC San Diego.

### Brain imaging data and phenotypes

Magnetic resonance imaging (MRI) data of the brains were collected for all subjects with various scanners. The imaging data were analyzed using FreeSurfer software and the cortical surface was reconstructed to measure surface areas at 160k surface locations for each hemisphere. To account for global effects, we divided the area measure of each location by the total surface area in each subject. The surface locations were then parceled into 12 regions and surface areas of each cortical region computed. The regions were previously defined using a data driven clustering technique that identified parcels of the human cortex maximizing their genetic correlations based on twin modeling [16]. The cortical surface areas were adjusted for age, gender, age-gender interaction, site effects, imaging device, the study cohort, and diagnosis where applicable. The phenotypes were also adjusted for the first ten eigenvectors of the genetic relationship matrix for the C5C.

### Genotype data

All subjects from C5C were genotyped with different commercial arrays. Genotypes from each sub-study were imputed separately with European panels from the 1000 Genome Project. After quality control and removal of related individuals and individuals of non-European ancestry, 2364 subjects with 2,480,482 genome-wide imputed variants from the C5C remained for subsequent analyses.

In this final combined cohort, 52% of the individuals were female; the subjects were aged 47 ± 24 y (range = [3, 90]); and 273, 128, 131, 147, and 66 subjects were diagnosed with mild cognitive impairment (MCI), Alzheimer’s disease (AD), schizophrenia (SCZ), bipolar disorder (BIP), and other psychosis (OP), respectively.

### Twin-based genetic correlations

The genetic correlations of surface area between cortical regions were derived using the classical twin modeling for the VETSA samples [16]. A standard bivariate twin AE model was used to estimate the proportion of phenotypic variance between cortical regions accounted for by additive genetic effects (A) and the individual-specific environmental effects (E) for each measure [11]. The structural equation modeling (SEM) application OpenMx was used to calculate and standardize the genetic covariance matrix yielding the genetic correlation matrix.

### Genotype-based genetic correlations

A standard bivariate variance component model [39] similar to the bivariate twin model was used to calculate genetic correlations of inter-regional cortical surface areas for the C5C. In the model, the phenotype was expressed as a linear function of the sum of additive genetic effects and the residual effects. But different from the twin model, the genetic component, in particular, the genetic relationship matrix, was estimated using the genotype data. The average information restricted maximum likelihood (AIREML) method as implemented in GCTA bivariate analysis [15] was used to carry out the estimates of the genetic correlations.

### Gene expression profiles and similarities

Transcriptomic data of six human brains at hundreds of anatomical locations was obtained from Allen Human Brain Atlas [17, 18]. There were one female and five males with an average age of 42.5. We first mapped the transcriptome locations to the locations used in the brain imaging analysis. The Allen brain atlas provided data in Montreal Neurological Institute (MNI) coordinates, which was used as the target space for our registration. We registered FreeSurfer space to MNI space by using FreeSurfer’s fsaverage T1 atlas, which was rigidly registered to an MNI T1 brain atlas (colin27). Fig 4 shows the resulting registration between FreeSurfer surface (fsaverage), MNI T1 atlas (colin27 displayed as slice) and Allen brain MNI coordinates displayed as a point cloud. After the registration, gene expression data defined at each point is mapped to FreeSurfer surface vertices by assigning each surface vertex the gene expression of the closest (Euclidean distance) Allen brain atlas coordinate using nearest neighbor interpolation. The gene expression profiles of cortical regions were subsequently derived, and finally aggregated over multiple brain samples. The binary gene expressions (expressed or unexpressed) were used and the similarity between gene expression profiles of any two cortical regions was measured with Jaccard coefficient, which computed the fraction of the number of genes expressed in both cortical regions over the total number of genes expressed in at least one of the two regions.

Network analysis for frontal lobe genes were performed using GeneMANIA [40] and visualized in Cytoscape [41]. All frontal genes/transcripts excluding intergenic transcripts were included, along with additional 20 related genes selected by GeneMANIA. Co-expression, co-localization, protein-protein interaction, and pathways were all considered,. The functional annotations of transcripts were updated with the new reference genome database via the Re-Annotator software [42].

### Regression with errors-in-both-variables (EIV)

To account for errors in both twin-based and genotype-based estimates of genetic correlations, a linear regression with errors-in-both-variables (EIV) model [24, 43], instead of the standard regression model, was used to study the relationship between the two estimates. A total least square approach was taken to fit the data. The variance of each data point was determined by the variance from both variables and the linear model.

### Matrix correlation

Mantel test [19, 44] was used to compute correlations between two correlation or similarity matrices. It is a permutation test. Since the mutual independent assumption between elements do not hold for similarly matrices, the significant level of correlation measures therefore cannot be obtained directly from normal probability. We acknowledge however if there exists spatial auto-correlation, the significant levels estimated by Mantel tests could be potentially inflated [45]. Both regression with EIV model and Pearson’s correlation model were used in Mantel test for each pair of matrices. The resulting test coefficient corresponded to either the slope of regression with both matrices scaled to have the same variance, or the correlation coefficient.

Full details are given in S1 Text.

## Supporting Information

S1 Fig Linear regression of genetic correlation *r*_*g*_ of combined-5-cohort (C5C) based on GCTA onto genetic correlation of VETSA cohort based on the twin AE model using the errors-in-both-variables (EIV) model. EIV model took error in measurements into consideration and showed significant correlations between the two genetic correlations. Lettered data-points indicate pairs of cortical regions by numbers (see Fig 1B). Colors represent standard errors (SE) of GCTA *r*_*g*_ estimates, clipped at 1. The genetic correlations shown in the graph were original values without standardization. Where an overall correlation between the two sets of genetic correlations was observed, some pairs of regions exhibited strong correlation (or anti-correlation) consistently. For instance, the pairs opercularis and subcentral region and the superior temporal region (clusters 5 & 6) had high *r*_*g*_ from both sample sets, and the surface areas of the two regions were also highly correlated, suggesting that the genetic correlation is likely underlying the phenotypic correlation, which is also consistent with both regions belonging to the same human-specific subdivision involved in language. The occipital region (cluster 12) was consistently anti-correlated with regions in frontal lobe (clusters 1–5), while correlated with superior parietal region (cluster 10) and likely with precuneus region (cluster 11, although with large error in the GCTA estimate), both genetically and phenotypically, across both datasets (See also Figs 1 & 2).
(PDF)

S1 Table Comparison of correlation matrices between cortical region surface areas of C5C and VETSA cohorts. See also Fig 1A.
(DOCX)

S2 Table Genetic correlations between cortical regions of VETSA sample set estimated with twin analysis. See also Figs 1C & 2A.
(DOCX)

S3 Table Genetic correlations between cortical regions of C5C sample set estimated by genotype-based GCTA-bivariate. See also Fig 2B.
(DOCX)

S4 Table Gene expression profile similarities between cortical regions using Allen Human Brain Human Atlas. See also Fig 2C.
(DOCX)

S5 Table The majority of genes were expressed in the cortical surface areas of all four lobes of the brain. A small percentage of the genes were either distinctively expressed in the cortical surface areas of one lobe or co-expressed in multiple but not all four lobes of the brain, which are listed below. See also Fig 5. Color code indicates location: exon, intron, intergenic, up/downstream, unknown.
(DOCX)

S6 Table Genes used in the network analysis for the frontal lobe, including exonic, intronic, and intronic_ncRNA transcripts, but excluding intergenic transcripts. See also Fig 5C.
(XLSX)

S7 Table Functional pathways associated with genes distinctively expressed in the frontal lobe (FDR < 0.1). See Fig 5C and S8 Table for the genes in the network.
(DOCX)

S8 Table Genes in the network in Fig 5C associated with functional pathways. (XLSX)

S1 Text Supplemental materials and methods and consortium authors. (DOCX)
