# Supplementary figures and images for: Conservation of Distinct Genetically-Mediated Human Cortical Pattern
Source: PLoS Genet. 2016 Jul 26;12(7):e1006143. doi: 10.1371/journal.pgen.1006143 (PMC4961377; doi:10.1371/journal.pgen.1006143)

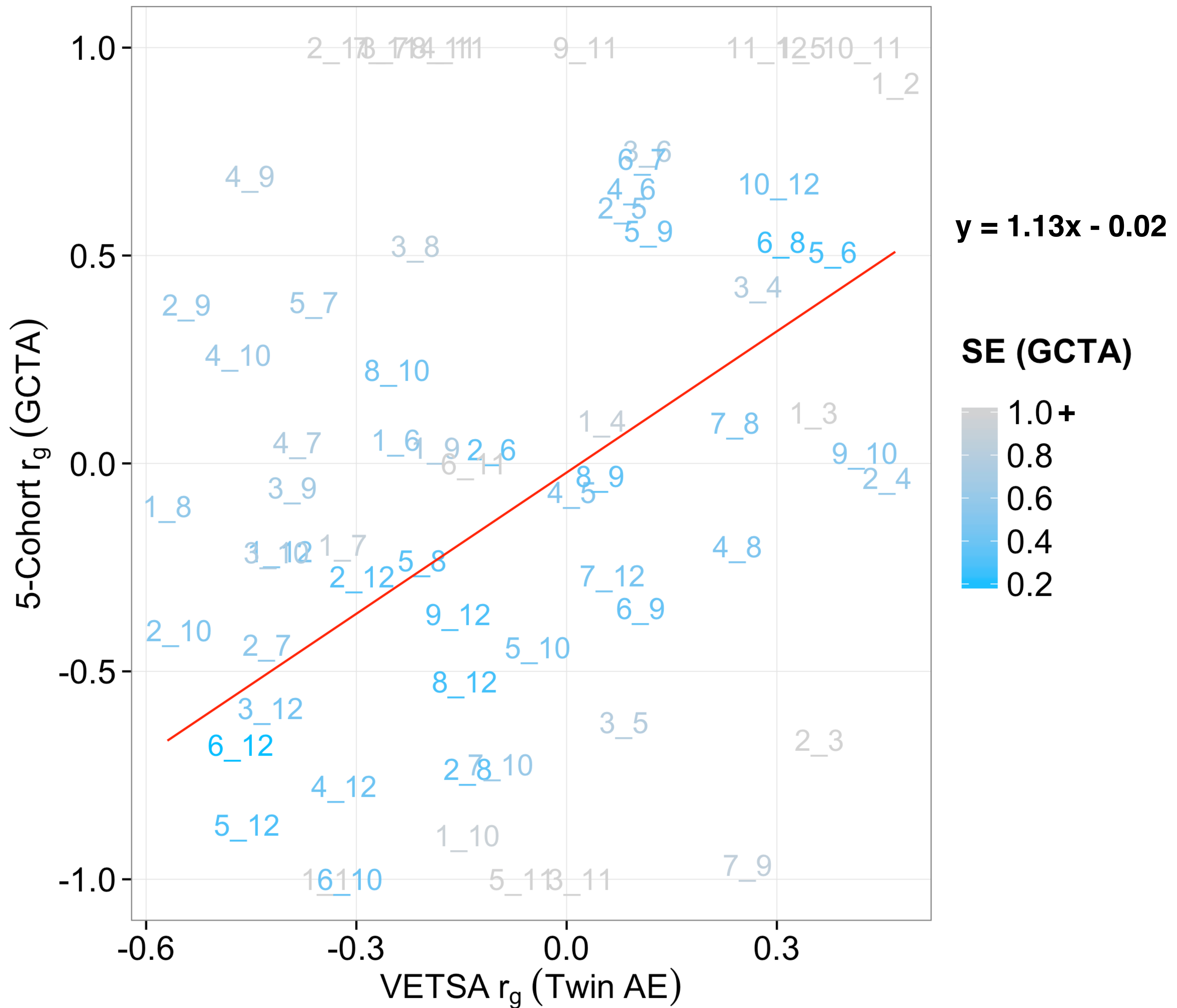

Supplement: S1 Fig — EIV model took error in measurements into consideration and showed significant correlations between the two genetic correlations. Lettered data-points indicate pairs of cortical regions by numbers (see Fig 1B). Colors represent standard errors (SE) of GCTA rg estimates, clipped at 1. The genetic correlations shown in the graph were original values without standardization. Where an overall correlation between the two sets of genetic correlations was observed, some pairs of regions exhibited strong correlation (or anti-correlation) consistently. For instance, the pairs opercularis and subcentral region and the superior temporal region (clusters 5 & 6) had high rg from both sample sets, and the surface areas of the two regions were also highly correlated, suggesting that the genetic correlation is likely underlying the phenotypic correlation, which is also consistent with both regions belonging to the same human-specific subdivision involved in language. The occipital region (cluster 12) was consistently anti-correlated with regions in frontal lobe (clusters 1–5), while correlated with superior parietal region (cluster 10) and likely with precuneus region (cluster 11, although with large error in the GCTA estimate), both genetically and phenotypically, across both datasets (See also Figs 1 & 2). (PDF) [file pgen.1006143.s001.pdf]
