# Supplementary material for: Conservation of Distinct Genetically-Mediated Human Cortical Pattern
Source: PLoS Genet. 2016 Jul 26;12(7):e1006143. doi: 10.1371/journal.pgen.1006143 (PMC4961377; doi:10.1371/journal.pgen.1006143)
Supplement: S1 Table — See also Fig 1A. (DOCX) [file pgen.1006143.s002.docx]

**S1 Table.** Comparison of correlation matrices between cortical region surface areas of C5C and VETSA cohorts. See also Fig 1A.

| *r*: C5C & VETSA* | 1 | 2 | 3 | 4 | 5 | 6 | 7 | 8 | 9 | 10 | 11 | 12 |
| --- | --- | --- | --- | --- | --- | --- | --- | --- | --- | --- | --- | --- |
| 1 motor premotor |  | 0.257 | 0.253 | -0.052 | 0.269 | -0.157 | -0.223 | -0.443 | -0.104 | -0.141 | -0.243 | -0.332 |
| 2 dorsolateral prefrontal | 0.165 |  | 0.194 | 0.402 | 0.017 | -0.147 | -0.324 | 0.016 | -0.506 | -0.429 | -0.263 | -0.288 |
| 3 dorsomedial frontal | 0.432 | 0.293 |  | 0.274 | 0.075 | 0.052 | -0.268 | -0.153 | -0.279 | -0.357 | 0.120 | -0.395 |
| 4 orbitofrontal | -0.099 | 0.332 | 0.216 |  | 0.014 | 0.095 | -0.444 | 0.261 | -0.390 | -0.416 | -0.057 | -0.324 |
| 5 pars opercularis & subcentral | 0.241 | 0.002 | -0.016 | 0.016 |  | 0.314 | -0.335 | -0.152 | 0.140 | -0.095 | 0.000 | -0.406 |
| 6 superior temporal | -0.126 | -0.127 | -0.005 | 0.115 | 0.294 |  | 0.001 | 0.067 | 0.139 | -0.172 | -0.002 | -0.419 |
| 7 posterolateral temporal | -0.302 | -0.258 | -0.225 | -0.239 | -0.251 | 0.200 |  | 0.019 | 0.210 | -0.047 | -0.256 | 0.083 |
| 8 anteromedial temporal | -0.352 | -0.280 | -0.253 | 0.125 | -0.163 | 0.226 | 0.184 |  | -0.103 | -0.283 | -0.169 | -0.087 |
| 9 inferior parietal | -0.138 | -0.330 | -0.194 | -0.238 | 0.135 | 0.074 | 0.058 | 0.004 |  | 0.340 | -0.015 | -0.118 |
| 10 superior parietal | -0.159 | -0.250 | -0.265 | -0.198 | -0.176 | -0.311 | -0.235 | -0.095 | 0.268 |  | 0.339 | 0.259 |
| 11 precuneus | -0.212 | -0.199 | -0.028 | -0.115 | -0.138 | -0.268 | -0.254 | -0.125 | -0.006 | 0.422 |  | 0.063 |
| 12 occipital | -0.344 | -0.234 | -0.439 | -0.259 | -0.307 | -0.405 | -0.054 | -0.060 | -0.266 | 0.060 | 0.143 |  |

* Lower triangular is of the combined-5-cohort; Upper triangular is of the VETSA cohort.
