# Supplementary material for: Conservation of Distinct Genetically-Mediated Human Cortical Pattern
Source: PLoS Genet. 2016 Jul 26;12(7):e1006143. doi: 10.1371/journal.pgen.1006143 (PMC4961377; doi:10.1371/journal.pgen.1006143)
Supplement: S2 Table — See also Figs 1C & 2A. (DOCX) [file pgen.1006143.s003.docx]

**S2 Table.** Genetic correlations between cortical regions of VETSA sample set estimated with twin analysis. See also Figs 1C & 2A.

| *r_g_* & SE* | 1 | 2 | 3 | 4 | 5 | 6 | 7 | 8 | 9 | 10 | 11 | 12 |
| --- | --- | --- | --- | --- | --- | --- | --- | --- | --- | --- | --- | --- |
| 1 motor premotor |  | 0.065 | 0.083 | 0.081 | 0.084 | 0.083 | 0.078 | 0.073 | 0.07 | 0.080 | 0.072 | 0.076 |
| 2 dorsolateral prefrontal | 0.469 |  | 0.089 | 0.078 | 0.076 | 0.079 | 0.080 | 0.067 | 0.08 | 0.088 | 0.078 | 0.072 |
| 3 dorsomedial frontal | 0.352 | 0.361 |  | 0.099 | 0.082 | 0.108 | 0.089 | 0.089 | 0.10 | 0.098 | 0.085 | 0.087 |
| 4 orbitofrontal | 0.050 | 0.457 | 0.274 |  | 0.073 | 0.091 | 0.076 | 0.075 | 0.09 | 0.082 | 0.068 | 0.093 |
| 5 pars opercularis & subcentral | 0.335 | 0.080 | 0.082 | 0.008 |  | 0.096 | 0.072 | 0.083 | 0.09 | 0.096 | 0.076 | 0.093 |
| 6 superior temporal | -0.243 | -0.106 | 0.116 | 0.093 | 0.380 |  | 0.093 | 0.094 | 0.10 | 0.102 | 0.098 | 0.085 |
| 7 posterolateral temporal | -0.319 | -0.427 | -0.260 | -0.384 | -0.361 | 0.108 |  | 0.076 | 0.09 | 0.093 | 0.067 | 0.088 |
| 8 anteromedial temporal | -0.570 | -0.141 | -0.215 | 0.244 | -0.206 | 0.307 | 0.240 |  | 0.09 | 0.094 | 0.082 | 0.089 |
| 9 inferior parietal | -0.187 | -0.542 | -0.391 | -0.452 | 0.117 | 0.106 | 0.258 | 0.048 |  | 0.096 | 0.096 | 0.091 |
| 10 superior parietal | -0.142 | -0.553 | -0.414 | -0.468 | -0.041 | -0.309 | -0.094 | -0.242 | 0.426 |  | 0.091 | 0.096 |
| 11 precuneus | -0.332 | -0.323 | 0.020 | -0.162 | -0.064 | -0.132 | -0.258 | -0.175 | 0.027 | 0.422 |  | 0.093 |
| 12 occipital | -0.409 | -0.292 | -0.422 | -0.317 | -0.456 | -0.465 | 0.066 | -0.145 | -0.155 | 0.303 | 0.285 |  |

* Lower triangular is genetic correlation *r_g_*; Upper triangular is standard error (SE).
