# Supplementary material for: Conservation of Distinct Genetically-Mediated Human Cortical Pattern
Source: PLoS Genet. 2016 Jul 26;12(7):e1006143. doi: 10.1371/journal.pgen.1006143 (PMC4961377; doi:10.1371/journal.pgen.1006143)
Supplement: S3 Table — See also Fig 2B. (DOCX) [file pgen.1006143.s004.docx]

**S3 Table.** Genetic correlations between cortical regions of C5C sample set estimated by genotype-based GCTA-bivariate. See also Fig 2B.

| *r_g_* & SE* | 1 | 2 | 3 | 4 | 5 | 6 | 7 | 8 | 9 | 10 | 11** | 12 |
| --- | --- | --- | --- | --- | --- | --- | --- | --- | --- | --- | --- | --- |
| 1 motor premotor |  | 1.015 | 1.102 | 0.918 | 1.148 | 0.561 | 0.908 | 0.564 | 0.736 | 0.942 | 97923.328 | 0.487 |
| 2 dorsolateral prefrontal | 0.915 |  | 1.237 | 0.601 | 0.511 | 0.349 | 0.557 | 0.352 | 0.600 | 0.479 | 13.838 | 0.316 |
| 3 dorsomedial frontal | 0.122 | -0.664 |  | 0.778 | 0.799 | 0.777 | 1.779 | 0.846 | 0.682 | 0.730 | 3.788 | 0.428 |
| 4 orbitofrontal | 0.102 | -0.035 | 0.425 |  | 0.504 | 0.469 | 0.754 | 0.469 | 0.769 | 0.635 | 674.410 | 0.396 |
| 5 pars opercularis & subcentral | 1.000 | 0.615 | -0.622 | -0.069 |  | 0.271 | 0.655 | 0.319 | 0.405 | 0.439 | 1.832 | 0.289 |
| 6 superior temporal | 0.056 | 0.034 | 0.751 | 0.661 | 0.508 |  | 0.464 | 0.246 | 0.370 | 0.387 | 0.000 | 0.182 |
| 7 posterolateral temporal | -0.196 | -0.436 | 1.000 | 0.050 | 0.388 | 0.733 |  | 0.461 | 0.879 | 0.619 | 11.726 | 0.442 |
| 8 anteromedial temporal | -0.102 | -0.735 | 0.524 | -0.200 | -0.233 | 0.533 | 0.098 |  | 0.353 | 0.413 | 1.589 | 0.266 |
| 9 inferior parietal | 0.037 | 0.382 | -0.058 | 0.691 | 0.559 | -0.349 | -0.966 | -0.030 |  | 0.501 | 9.202 | 0.290 |
| 10 superior parietal | -0.894 | -0.401 | -0.214 | 0.261 | -0.443 | -1.000 | -0.724 | 0.225 | 0.025 |  | 2.704 | 0.421 |
| 11 precuneus** | -1.000 | 1.000 | -1.000 | 1.000 | -1.000 | 0.000 | 1.000 | 1.000 | 1.000 | 1.000 |  | 1.382 |
| 12 occipital | -0.212 | -0.272 | -0.589 | -0.776 | -0.870 | -0.677 | -0.269 | -0.525 | -0.362 | 0.674 | 1.000 |  |

* Lower triangular is genetic correlation *r_g_*; Upper triangular is standard error (SE).

** GCTA either did not converge or converged with vary large errors, rendering these estimates completely unreliable.
