# Supplementary material for: Conservation of Distinct Genetically-Mediated Human Cortical Pattern
Source: PLoS Genet. 2016 Jul 26;12(7):e1006143. doi: 10.1371/journal.pgen.1006143 (PMC4961377; doi:10.1371/journal.pgen.1006143)
Supplement: S4 Table — See also Fig 2C. (DOCX) [file pgen.1006143.s005.docx]

**S4 Table.** Gene expression profile similarities between cortical regions using Allen Human Brain Human Atlas. See also Fig 2C.

| Jaccard coefficient | 1 | 2 | 3 | 4 | 5 | 6 | 7 | 8 | 9 | 10 | 11 |
| --- | --- | --- | --- | --- | --- | --- | --- | --- | --- | --- | --- |
| 1 motor premotor |  |  |  |  |  |  |  |  |  |  |  |
| 2 dorsolateral prefrontal | 0.9884 |  |  |  |  |  |  |  |  |  |  |
| 3 dorsomedial frontal | 0.9894 | 0.9882 |  |  |  |  |  |  |  |  |  |
| 4 orbitofrontal | 0.9827 | 0.9863 | 0.9886 |  |  |  |  |  |  |  |  |
| 5 pars opercularis & subcentral | 0.9897 | 0.9904 | 0.9881 | 0.9855 |  |  |  |  |  |  |  |
| 6 superior temporal | 0.9857 | 0.9855 | 0.9894 | 0.9894 | 0.9882 |  |  |  |  |  |  |
| 7 posterolateral temporal | 0.9842 | 0.9801 | 0.9854 | 0.9852 | 0.9823 | 0.9895 |  |  |  |  |  |
| 8 anteromedial temporal | 0.9752 | 0.9735 | 0.9796 | 0.9839 | 0.9747 | 0.9827 | 0.9821 |  |  |  |  |
| 9 inferior parietal | 0.9878 | 0.9814 | 0.9855 | 0.9826 | 0.9847 | 0.9873 | 0.9904 | 0.9793 |  |  |  |
| 10 superior parietal | 0.9851 | 0.9783 | 0.9843 | 0.9804 | 0.9815 | 0.9844 | 0.9899 | 0.9783 | 0.9919 |  |  |
| 11 precuneus | 0.9803 | 0.9738 | 0.9813 | 0.9794 | 0.9768 | 0.9823 | 0.9892 | 0.9796 | 0.9881 | 0.9905 |  |
| 12 occipital | 0.9770 | 0.9715 | 0.9764 | 0.9760 | 0.9750 | 0.9794 | 0.9842 | 0.9765 | 0.9842 | 0.9872 | 0.9874 |
