# Supplementary material for: Conservation of Distinct Genetically-Mediated Human Cortical Pattern
Source: PLoS Genet. 2016 Jul 26;12(7):e1006143. doi: 10.1371/journal.pgen.1006143 (PMC4961377; doi:10.1371/journal.pgen.1006143)
Supplement: S5 Table — A small percentage of the genes were either distinctively expressed in the cortical surface areas of one lobe or co-expressed in multiple but not all four lobes of the brain, which are listed below. See also Fig 5. Color code indicates location: exon, intron, intergenic, up/downstream, unknown. (DOCX) [file pgen.1006143.s006.docx]

**S5 Table.** The majority of genes were expressed in the cortical surface areas of all four lobes of the brain. A small percentage of the genes were either distinctively expressed in the cortical surface areas of one lobe or co-expressed in multiple but not all four lobes of the brain, which are listed below. See also Fig 5. Color code indicates location: exon, intron, intergenic, up/downstream, unknown.

| Lobes | #genes | Distinctively expressed genes (a consensus list) |
| --- | --- | --- |
| Frontal | 153 | CD1B:exon COL8A2:exon CYP1A1:exon EGF:exon FBP1:exon GBP1:exon FMO4:exon HOXA5:exon HLA-DQA2:exon KRT5:exon LAIR2:exon IL18:exon MN1:exon STAT5A:exon SLC34A1:exon TDO2:exon MTL5:exon TSPAN2:exon ABCC4:exon HSF2BP:exon CLCF1:exon FBXW8:exon ROPN1:exon CCDC169-SOHLH2/SOHLH2:exon SLC4A5:exon RSG1:exon BORA:exon TMEM27:exon GSDMD:exon RBP5:exon PLA2G12B:exon DPP9:exon CLRN3:exon DEPDC4:exon DEFB104B/DEFB104A:exon AK7:exon LSMEM2:exon C1orf65:exon SLC9B1:exon TMEM31:exon GAS2L3:exon OR13J1:exon KIF7:exon UVSSA:exon TNN:exon TRIM6:exon LRIT2:exon FAM3B:exon PTPN6:exon DLX5:exon GRM6:exon TGFB1I1:exon ANKRD31:exon SLC13A2:exon ZC3H12D:exon CPS1:exon LINC00638:exon LOC728819:exon PTGES2-AS1:exon BTBD19:exon DHRS7C:exon SMAD5-AS1:exon CALHM3:exon PGPEP1L:exon SLC26A9:exon CNGA3:exon BMP3:exon PQLC2:exon ATP6V1C2:exon RAB27A:exon ICAM1:exon HBE1:exon CHRNE:exon UCA1:exon PLCB2:exon LOC100507377:exon TRPC2:exon GLUD1P7:exon TSPY1/2/3/4/8/10:exon NUTM2A/B/D:exon FBXW10/CDRT1:exon F11-AS1/F11:exon F2RL3/CPAMD8:exon CYP4F12/CYP4F24P:exon APOBEC3F/G:exon HCG22:exon/ncRNA_exon MCCD1:exon BIRC5:exon GJD4:exon/intron MXRA5/NONE-GYG2P1:exon/intergenic SAP30/HMGB2-SAP30/SAP30-SCRG1:exon/intergenic C7orf55-LUC7L2/LUC7L2:intron CCDC97:intron FOXO1:intron FOXN3:intron SLC39A9:intron TLX1NB:intron CNIH4:intron SRGN:intron RAB8B:intron DESI2:intron FAM107B:intron DMXL1:intron PLEKHA7:intron SMURF2:intron MAP3K8:intron LDLR:intron LOC100287944/LOC100505978:ncRNA_intron PRR26:ncRNA_intron DPY19L2P1/2:ncRNA_intron HIST2H2BF/FCGR1C-PPIAL4B:intron/intergenic AMOT-HTR2C:intergenic FAM86C2P-UNC93B1:intergenic CCDC36-C3orf62:intergenic RAB3IP-CNOT2:intergenic SLC35G4-GNAL:intergenic AR-OPHN1:intergenic ZCCHC3-SOX12:intergenic CNPY1-RBM33:intergenic LINC00865-HTR7:intergenic OR4E2-DAD1:intergenic CALHM1-CALHM3:intergenic SCRG1-HAND2:intergenic BCHE-ZBBX:intergenic NONE-SLC39A10:intergenic GLCE-PAQR5:intergenic METTL2B-LINC01000:intergenic ZNF503-AS2-C10orf11:intergenic PRDM5-NDNF:intergenic GRXCR1-KCTD8:intergenic BRI3BP-AACS:intergenic ADAMTS2-RUFY1:intergenic PNMA5-PNMA3:intergenic SOX6-C11orf58:intergenic CDS2-PROKR2:intergenic MIR548I2-DRD5:intergenic NONE-GRIA3:intergenic TTC26-UBN2:intergenic EHD3-XDH:intergenic FAM74A1-SPATA31A3/LOC643648-NONE/SPATA31A5-PTGER4P2-CDK2AP2P2:intergenic ZNF839-CINP:intergenic ZNF267-HERC2P4/RNU6-76P-LINC00273/SLC6A10P-TP53TG3C:intergenic KIR2DL2-KIR2DL4/KIR2DL1-LOC100287534/LOC100287534:intergenic/intron PPFIA2:downstream B4GALNT3:downstream ACAT1:downstream/intron CDCP2/CDCP2-CYB5RL:downstream/intergenic A_24_P924114 A_23_P112957 A_24_P450398 A_24_P458168 A_24_P910381 A_32_P211765 |
| Temporal | 20 | CD70:exon TNFRSF11A:exon KL:exon PSD4:exon NXF5:exon TPH2:exon ASB9:exon EXD1:exon PAX2:exon KCNJ1:exon TRIM71:exon PYDC2:exon AQP3:exon LOC643733:exon RNASET2:intron WDFY4:intron PABPC1-YWHAZ:intergenic CWH43-NONE:intergenic ANKRD20A12P-LOC100130000:intergenic ZNF766:upstream |
| Parietal | 3 | TINAGL1:exon LOC100130700:exon FAM3C-PTPRZ1:intergenic |
| Occipital | 30 | POTEB/POTEB2/POTEC/POTED/LOC100288966:exon IRF1:exon CEACAM7:exon HAS3:exon IFNA16:exon SPAG6:exon VCX/VCX2/VCX3A/VCX3B:exon LHX9:exon REXO1:exon APOL5:exon TTC29:exon GPR63:exon RNF148:exon SCN5A:exon CNKSR1:exon C16orf46:exon FAM196B:exon SPDYE7P:exon COBLL1:exon PCDHA1-10:intron SIK3:intron TENM2:intron CACNA1C:intron ZNF397:intron LOC729911:ncRNA_intron SF1/CDC42BPG: intron/downstream FAIM-PIK3CB:intergenic ZSCAN5A-ZNF542:intergenic LINC00202-1-ANKRD26:intergenic S100P-MRFAP1L1:intergenic |
| Frontal + Temporal | 108 | LIMD1:exon CPVL:exon GRAMD2:exon SCN7A:exon ZNF844-ZNF788/ZNF878:intergenic/intron PBLD/ATOH7-PBLD:intron/intergenic STT3B/GADL1-STT3B:upstream/intergenic SIAH3:downstream LOC100506804:downstream RAD51C:downstream COL1A1:exon GPX2:exon GUCY2F:exon IGFBP4:exon PTGER4:exon SPRR3:exon TRPC6:exon TTPA:exon IL18R1:exon GALR2:exon PROM1:exon GPRIN2:exon GPR160:exon GNMT:exon CIDEB:exon DUSP13:exon TAF7L:exon GBA3:exon SAMSN1:exon CBX8:exon CES3:exon DCSTAMP:exon SIGLEC10:exon C19orf40:exon SYTL1:exon TWIST2:exon KIR3DL3:exon DNAAF1:exon LYPD6B:exon UBQLNL:exon CCBE1:exon ARHGAP36:exon AKAP14:exon DENND2C:exon WDR49:exon DLEU7:exon THEM5:exon FREM2:exon SLC6A18:exon SLCO4C1:exon C12orf42:exon KCNE3:exon SPRR2G:exon BCL2L15:exon RUNX1:exon SMPDL3B:exon EGFLAM:exon C1QL4:exon CCNI2:exon CAPSL:exon SCN7A:exon PDCD4-AS1:exon SHBG:exon SCIN:exon LOC100288846:exon PACRGL:exon LINC00671:exon IQCF3:exon ZDHHC15:exon BHMT:exon LINC01013:exon FFAR4:exon LINC00578:exon OR13C5/OR13C2:exon RPS6KB1/MED13-TBC1D3P2:exon/intergenic LOC730227/CHIT1-LOC730227:exon/intergenic ACSM5/ACSM2B-ACSM1:exon/intron/intergenic/downstream TDRD12/SLC7A9:exon/downstream GSG2/ITGAE:exon/intron AKT2:intron FAM120B:intron CEP89:intron UBR4:intron MNS1/TEX9:intron EIF2AK4:intron FDPS:intron NMNAT3:intron ZFHX4:intron CSGALNACT1:intron PTGER4P2-CDK2AP2P2-LOC286297/CNTNAP3B-LOC643648:intergenic PNP-RNASE10:intergenic PARS2-TTC22:intergenic MBOAT1-E2F3:intergenic LOC339568-MAFB:intergenic SLC2A9-WDR1:intergenic OPRK1-ATP6V1H:intergenic COLEC10-MAL2:intergenic DOK6-CD226:intergenic ZCCHC2-PHLPP1:intergenic FAM214A-ONECUT1:intergenic GABRQ-MAGEA6:intergenic FOCAD-PTPLAD2:intergenic C1QTNF8-CACNA1H:intergenic LRBA-RPS3A:intergenic NONE-NONE/LRRC37A3:intergenic/intron MAN2C1/SIN3A:upstream/downstream RP13-16H11.1 A_23_P348587 |
| Frontal + Parietal | 44 | FCGR3B:exon FMO1:exon PLD2:exon PTH2R:exon RPL3L:exon FRMPD1:exon TTC40:exon ABCG5:exon KRTAP4-4:exon C1QTNF7:exon RNF113B:exon TEX26:exon AMDHD1:exon C5orf64:exon LINC00545:exon METRNL:exon CCNE2:exon UBASH3B:exon METTL21C:exon TMEM184A:exon MBOAT1:exon SKAP1:exon PLA2G2C:exon CCDC150:exon C22orf34:exon CACTIN-AS1:exon/ncRNA_intron ANK3:intron CD22:intron LOC731779-GPR20:intergenic ZNF749-VN1R1:intergenic IPCEF1-CNKSR3:intergenic ITGB8-ABCB5:intergenic CLYBL-ZIC5:intergenic REXO1L2P-PSKH2:intergenic SENP2-IGF2BP2:intergenic NOX1-XKRX:intergenic LOC643648-FAM27C/LOC642929-SPATA31A6/NONE-NONE/ZNF658-SPATA31A4:intergenic SOGA2-NDUFV2:intergenic SH3BGRL-POU3F4:intergenic NMUR1-C2orf57:intergenic A_24_P16361 A_24_P273074 A_24_P564761 A_24_P920664 |
| Frontal + Occipital | 24 | CCNA2:exon FOXD2:exon HAS1:exon NTF4:exon SLC16A4:exon HHLA2:exon DCD:exon DCAF4L2:exon ZNF366:exon YIPF7:exon PCDHGA11:exon BCOR:exon METTL20:exon GPRIN3:exon MAML3:exon OSBPL9:exon FUT9:intron CIITA:intron GARNL3/FAM129B:intron LOC646743/TISP43:ncRNA_intron TRH-ALG1L2:intergenic CCDC57-SLC16A3:intergenic TMEM178B/MRPS33-TMEM178B:upstream/intergenic A_24_P230486 |
| Temporal + Parietal | 1 | AOX1:exon |
| Temporal + Occipital | 1 | RSPH10B/RSPH10B2:exon |
| Parietal + Occipital | 19 | HTR3C:exon ADAMDEC1:exon RHOXF1:exon DQX1:exon PCDHA6/PCDHA8:exon OR2M4:exon CAPN14:exon SGCG:exon C20orf166-AS1:exon/ncRNA_intron CUX1:intron FAM19A2:intron GNG7:intron ESAM:intron STMN3:intron CCNB2:intron NONE-NONE:intergenic TEX29-SOX1:intergenic MIOS:downstream A_32_P99399 |
| Temporal + Parietal + Occipital | 4 | CGB1:exon ADAM6-LINC00226/LINC00221-NONE:intergenic LINC00221-NONE:intergenic A_32_P200586 |
| Frontal + Parietal + Occipital | 35 | CRHR2:exon DPP4:exon STK3:exon STC2:exon DKK1:exon EHF:exon SIX4:exon LRRC48:exon ZC3H12A:exon FAM83D:exon PXDNL:exon OR10A4:exon OR10K1:exon C2orf62:exon TEAD4:exon PCDHGB3:exon MIR31HG:exon LINC00574:exon HAUS5:exon LRRC9:exon TDH:exon/ncRNA_intron C7orf55-LUC7L2/LUC7L2:intron CACNG2:intron ARID4B:intron HIVEP2:intron TBK1:intron ANKS6:intron LRPPRC:intron FAM81B/TTC37/FAM81B-TTC37:intron/intergenic LINC01004-KMT2E-AS1:intergenic ELTD1-LPHN2:intergenic EPHA1-AS1-CTAGE15/CTAGE6-LOC154761:intergenic GOLGA8CP-NBEAP1:intergenic GCKR-C2orf16:intergenic A_24_P677712 |
| Frontal + Temporal + Occipital | 22 | CERKL/ITGA4:exon GML:exon IRAK2:exon OGN:exon PPY:exon DOK2:exon EGFL6:exon SLC30A6:exon RGS8:exon PUS10:exon GPR133:exon DNAJB13:exon OR52B6:exon DCLK3:exon LINC00894:exon CACNA1B:exon DKFZP434I0714:exon FLJ42393:exon HTR1D:exon GABRP:exon TMCO1:upstream EIF2B5:upstream |
| Frontal + Temporal + Parietal | 138 | CBLN1:exon HOXC12:exon HCK:exon KRT7:exon KRT33B:exon LGALS2:exon LGALS9:exon MMP14:exon MPO:exon MSH5/MSH5-SAPCD1:exon NEK2:exon PDE6H:exon PRTN3:exon RAD51:exon RXRG:exon XIST:exon PRSS12:exon DDO:exon ADAMTS3:exon TOM1L1:exon IGSF6:exon LYVE1:exon CD300A:exon LILRA4:exon NUDT13:exon OR8B8:exon DMGDH:exon TLR7:exon CD244:exon DCHS2:exon MYO3A:exon TMEM40:exon HRASLS2:exon MRAP:exon MEPE:exon NXN:exon RGS18:exon KLHL1:exon HKDC1:exon ELL3:exon GAL3ST4:exon GSG1:exon CDCA7:exon C10orf11:exon DYDC2:exon PRAP1:exon PIEZO2:exon OSR1:exon PLEKHA7:exon DAW1:exon ALS2CR11:exon IL20RB:exon TRIML2:exon HIST1H2AA:exon FERD3L:exon KRTAP19-7:exon TUSC5:exon LHX8:exon OR4A47:exon KIAA0101:exon UNG:exon TGFB1:exon TTLL9:exon FGL1:exon RAD51B:exon ANAPC2:exon PIRT:exon LOC286467:exon CYP4F11:exon HPGD:exon SLC18A1:exon CHEK1:exon DPEP1:exon PLAC8:exon CES5A:exon FAM110C:exon SVOPL:exon SNAP25-AS1:exon ACADS:exon SLC27A6:exon CXCL1:exon DPYS:exon PIK3CG:exon FLJ35934:exon PLXNC1:exon WNK3:exon LINC01094:exon IQCF4:exon PITPNM3:exon PTF1A:exon GYPA/GYPE/GYPB:exon NPHS1/PRODH2:exon ANKRD55:exon/intron AP5Z1/RADIL-MMD2:exon/intergenic GPN3:intron KIAA1755:intron AFG3L2:intron ST18:intron RGS5:intron C3:intron ELMO1:intron ART3:intron TAT/ZNF23-ZNF19:intron/intergenic LOC100507412-RNA5-8S5:intergenic SMEK1-CATSPERB:intergenic LINC00467-RD3:intergenic BOLA3-AS1-MOB1A:intergenic NEFH-THOC5:intergenic BLACE-EN2:intergenic OR7E156P-NONE:intergenic PCDH11X-NAP1L3:intergenic BOD1-CPEB4:intergenic ANKH-FBXL7:intergenic AGAP11-FAM25A:intergenic MIR4514-MESDC1:intergenic OPCML-SPATA19:intergenic NR2F2-SPATA8-AS1:intergenic ASB7-ALDH1A3:intergenic GRXCR1-KCTD8:intergenic SLC38A2-SLC38A4:intergenic OSR1-LINC00954:intergenic NONE-GYG2P1:intergenic PIH1D3-PRPS1:intergenic C4orf27-LOC100506085:intergenic AASS-FEZF1:intergenic C21orf62-OLIG2:intergenic TMEM252-PIP5K1B/PGM5-TMEM252:intergenic CD99P1-CD99/CD99-XG/CD99-XGPY2:intergenic RNF24:downstream GJD3:upstream A_32_P69333 A_24_P118813 A_23_P7719 A_24_P255763 A_24_P460195 A_24_P59239 A_24_P861869 A_24_P911973 |
