# Supplementary material for: Conservation of Distinct Genetically-Mediated Human Cortical Pattern
Source: PLoS Genet. 2016 Jul 26;12(7):e1006143. doi: 10.1371/journal.pgen.1006143 (PMC4961377; doi:10.1371/journal.pgen.1006143)
Supplement: S7 Table — See Fig 5C and S8 Table for the genes in the network. (DOCX) [file pgen.1006143.s008.docx]

**S7 Table.** Functional pathways associated with genes distinctively expressed in the frontal lobe (FDR < 0.1). See Fig 5C and Table S8 for the genes in the network.

| Function | FDR | Genes in network | Genes in genome |
| --- | --- | --- | --- |
| interferon-gamma-mediated signaling pathway | 3.244E-04 | 8 | 73 |
| cellular response to interferon-gamma | 7.846E-04 | 8 | 89 |
| response to interferon-gamma | 1.901E-03 | 8 | 105 |
| clathrin-coated endocytic vesicle membrane | 1.148E-02 | 5 | 35 |
| clathrin-coated endocytic vesicle | 1.816E-02 | 5 | 40 |
| transport vesicle membrane | 4.195E-02 | 5 | 49 |
| MHC class II receptor activity | 5.397E-02 | 3 | 11 |
| lymphocyte costimulation | 5.397E-02 | 5 | 61 |
| endocytic vesicle membrane | 5.397E-02 | 6 | 95 |
| ER to Golgi transport vesicle membrane | 5.397E-02 | 4 | 31 |
| transport vesicle | 5.397E-02 | 6 | 92 |
| coated vesicle membrane | 5.397E-02 | 6 | 100 |
| T cell costimulation | 5.397E-02 | 5 | 61 |
| regulation of cell activation | 5.397E-02 | 9 | 254 |
| regulation of lymphocyte activation | 6.084E-02 | 8 | 208 |
| positive regulation of lymphocyte activation | 6.285E-02 | 7 | 155 |
| ER to Golgi transport vesicle | 6.958E-02 | 4 | 35 |
| clathrin-coated vesicle membrane | 6.958E-02 | 5 | 68 |
| positive regulation of leukocyte activation | 8.162E-02 | 7 | 166 |
| regulation of T cell activation | 8.676E-02 | 7 | 169 |
| regulation of leukocyte activation | 8.910E-02 | 8 | 232 |
| positive regulation of cell activation | 8.910E-02 | 7 | 172 |
| endocytic vesicle | 9.382E-02 | 7 | 175 |
