# Supplementary material for: Conservation of Distinct Genetically-Mediated Human Cortical Pattern
Source: PLoS Genet. 2016 Jul 26;12(7):e1006143. doi: 10.1371/journal.pgen.1006143 (PMC4961377; doi:10.1371/journal.pgen.1006143)
Supplement: S1 Text — (DOCX) [file pgen.1006143.s010.docx]

**Supplemental Materials and Methods**

**S1. Participants**

The samples for the twin analysis was part of the *Vietnam Era Twin Study of Aging (VETSA) study* (1). There were 466 participants, of which 99 pairs were dizygotic twins and 134 pairs monozygotic twins. The sample is representative of U.S. middle-aged men in their demographic and health characteristics. A combined sample of five sub-study cohorts (referred to as combined-5-cohort or C5C) is made of 605 subjects from the Thematically Organized Psychosis (TOP) study, 842 Health Study of Nord-Trøndelag (HUNT) subjects, 325 Norwegian Cognitive Neuro-Genetics (NCNG) subjects, 726 Alzheimer’s Disease Neuroimaging Initiative (ADNI) subjects, and 1198 Pediatric Imaging Neurocognition and Genetics (PING) subjects. They are detailed as following.

*Thematically Organized Psychosis (TOP) Subjects.* Data from 1579 subjects from the TOP study were analyzed. MRI data was available for 605 of the subjects, including 236 controls, 144 subjects with schizophrenia, 157 subjects with bipolar disorder (BD), and 68 subjects diagnosed with other psychotic disorder not otherwise specified. Fifty percent of these subjects were women; the subjects were aged 35 ± 11 y (range=[17, 70]). Genotyping: DNA was genotyped on the Affymetrix 6.0 array. All subjects self-reported Norwegian ancestry. Principal components analysis (PCA) of an allele-sharing distance matrix across all subjects suggested one non-European ancestry genetic outlier. Brain imaging: MRI scans were performed with a 1.5 T Siemens Magnetom Sonata scanner equipped with a standard head coil.

*Health Study of Nord-Trøndelag (HUNT) Subjects.* Data from 905 subjects from the HUNT study were analyzed. Usable cortical MRI data was available for 842 of the subjects. Fifty-three percent of the subjects were women; the subjects were aged 58 ± 4 y (range=[50, 66]). Genotyping: DNA was genotyped on the Illumina Omni 2.5M BeanChip array. All subjects were recruited in Norway. PCA of an allele-sharing distance matrix across all subjects suggested 2 non-European ancestry genetic outliers. Brain imaging: MRI scans were per- formed with a General Electric Signa HDx 1.5 T scanner.

*Norwegian Cognitive NeuroGenetics (NCNG) Subjects.* Data from 670 subjects from the NCNG study were analyzed. Usable MRI data was available for 325 of the subjects. Sixty-nine percent of the subjects were women; the subjects were aged 52 ± 17 y (range=[19, 79]). Genotyping: DNA was genotyped with the Illumina Human610-Quad BeadChip. All subjects self-reported Norwegian ancestry. PCA of an allele- sharing distance matrix across all subjects did not suggest any non-European ancestry genetic outliers. Brain imaging: Participants have gone through a standard structural MRI protocol optimized for morphometric analysis. Further details on MRI acquisition and protocol are available in Espeseth et al., 2012 (2).

*Alzheimer’s Disease Neuroimaging Initiative (ADNI) Subjects.* Data from 793 subjects from the ADNI database were analyzed (http://adni.loni.usc.edu). Usable MRI data was available for 726 of the subjects. Subjects who self-reported as white and non-Hispanic included 161 individuals with Alzheimer’s Disease (AD) and 216 individuals with Mild Cognitive Impairment (MCI), and 216 subjects as controls. Forty-three percent of the subjects were women; the subjects were aged 75 ± 7 y (range = [55, 92]). Genotyping: DNA was genotyped with the Illumina Human610-Quad BeadChip. PCA of an allele-sharing distance matrix was used to remove 85 individuals as non-European ancestry genetic outliers. Brain Imaging: MRI data were collected on 1.5-T scanners at many study centers across the United States. Raw MR images were downloaded from the ADNI data page of the public ADNI site at the LONI website published in 2007.

*Pediatric, Imaging, Neurocognition, and Genetics (PING) Subjects.* Data from 1406 subjects were obtained from the PING database (http://ping.chd.ucsd.edu/). Usable MRI data for 1198 subjects were included. Forty-eight percent of the subjects were female; the subjects were aged 12 ± 5 y (range=[3, 21] y). Genotyping: DNA was genotyped with the Illumina Human660W-Quad BeadChip. Based on PCA of an allele-sharing distance matrix, 668 individuals were re- moved as non-European ancestry genetic outliers. Brain Imag- ing: T1-weighted MRI data were collected on 3-T scanners at nine study centers across the United States. Specific MRI scanner protocols are available at the PING study website (<http://ping.chd.ucsd.edu/>).

A total of 5353 subjects were analyzed; data were included from 3696 subjects with available and sufficient quality MRI scans for subsequent analyses (over half of the excluded datasets only had genotypes collected).

**S2. Genotype quality control**

All studies were genotyped using different commercial arrays. Standard genome-wide association quality control measures were applied to each study individually using the PLINK toolset (3, 4). Samples missing more than 5% of SNPs, with a minor allele frequency less than 1%, or failing a test of Hardy-Weinberg equilibrium (p < 10−6), were excluded. Individual samples showing an over- or under-abundance of heterozygosity (>5 standard deviations from the mean) were labeled as poor quality and also excluded from subsequent analyses (5). Furthermore, to ensure all individuals were unrelated, functions available in the software package Genome-wide Complex Trait Analysis (GCTA) (6) were used to estimate kinship coefficients from SNP genotypes for all pairs of individuals in the combined cohort. Population stratification and ancestry were assessed against a reference sample consisting of individuals from the HapMap III (7) and 1000 Genomes (8) via PCA implemented in the software package EIGEN- SOFT (9). One half of each pair of individuals with an estimated relatedness greater than 0.025 or 0.1 were excluded (10). Using the more stringent threshold at 0.025, 575 individuals were removed, leaving a total of 2364 individuals for the subsequent analyses. In this combined cohort of European ancestry with minimal relatedness between subjects [values in genetic relationship matrix (GRM) < 0.025], 52% of the individuals were female; the subjects were aged 47 ± 24 y (range = [3, 90]); and 273, 128, 131, 147, and 66 subjects were diagnosed with mild cognitive impairment (MCI), Alzheimer’s disease (AD), schizophrenia (SCZ), bipolar disorder (BIP), and other psychosis (OP), respectively. While a less stringent threshold of the estimated relatedness may provide a greater power with a larger sample size (2698), a more stringent threshold is less susceptible to potential confounding from cryptic relatedness for subsequent analyses. We thus reported the results from the set of sample using the threshold of the estimated relatedness at 0.025.

**S3. Genotype imputation**

To maximize information present in the data and allow for comparison across multiple samples genotyped on different platforms, genotype imputation was performed using the software packages MaCH (11) and Minimac (12). Reference data included datasets available at the time of imputation, such as resulting from the 1000 Genomes Project (13). A quality control metric (r2) was provided by Minimac and a threshold of r2 > 0.5 was used to declare successful imputation. TOP, HUNT, NCNG, ADNI, and PING genotypes were independently merged with reference population from the sequencing by the 1,000 Genomes Project. MACH 1.0 was used to impute genotypes with the default settings; only SNPs that passed imputation quality control (R > 0.5) were included for additional analysis.

**S4. MRI processing**

MRI scans were analyzed with software developed at the University of California at San Diego, Multi-Modal Imaging Laboratory based on the freely available FreeSurfer software package (http://freesurfer-software.org/). The cortical surface was reconstructed to measure surface areas at each surface location (a total of over 160,000 locations for each hemisphere) using a semi-automated approach (14, 15). Variation in image intensity due to magnetic field inhomogeneities was corrected, a normalized intensity image was created, and the skull (non-brain) was removed from this image. The resulting surface was covered with a polygonal tessellation and smoothed to reduce metric distortions. A refinement procedure was then applied to obtain a representation of the gray/white boundary, and the resulting surface was subsequently deformed outwards to obtain an explicit representation of the pial surface. Once generated, the cortical surface model was manually reviewed and edited for technical accuracy for some datasets. Maps were spatially smoothed and placed into a common coordinate system using a non-rigid high-dimensional spherical averaging method to align cortical folding patterns (15). Due to the standardized procedure for image acquisition and analysis, the MRI data consistency was maximized for pooling the data across studies.

**S5. Genetically based cortical surface area phenotypes**

We previously used a data driven fuzzy clustering technique to identify parcels of the human cortex that are maximally genetically correlated (i.e., under control of the same genetic factors) based on the MRI scans of over 400 twins (16, 17). We used this twin-based cluster maps to assign a parcellation label to each location on a cortical surface based on partial membership information estimated from the clustering analysis and calculated the weighted average surface area within each region for each subject. We used these regions, defined a priori on the basis of genetic information, to increase power for detecting effects and minimize multiple comparisons after reducing image dimensionality to these parcels.

To account for global effects, we divided the area measure of each location by the total surface area, so that the observed effects were specific to region of interest rather than having global associations with total surface area (16, 17). In the present study, in all analyses we further adjusted each phenotype for the covariates of age, gender, age-gender interaction, scanner, diagnosis, study cohort, and the first ten eigenvectors of the GRM where applicable. The sample age ranged from 3 to 90. We used polynomial basis functions and a Generalized Additive Modeling (GAM) framework to model age effects as nonparametric smooth functions to control for considerable age disparities (18), so the effects that we characterized could be generalized across the lifespan.

**S6. Phenotypic correlations**

The phenotypic correlation between two cortical regions is defined as the phenotypic covariance between those regions divided by the product of the standard deviations for each of the two regions. To compute the phenotypic correlations from the VETSA cohort, we used only one randomly chosen individual from each twin pair.

**S7. Effective number of independent phenotypes**

It has been shown that the total amount of correlation among a set of variables can be measured by the variance of the eigenvalues (λ_s_) derived from the correlation matrix (19, 20). Higher correlation among variables leads to higher variance of λs. Let M be the number of variables represented in the correlation matrix, then the variance of λs will range between zero, when all variables are independent, and M, when collective correlation is at its maximum. The proportional reduction of the number of variables can therefore be estimated by the ratio of the observed eigenvalue variance (Var(λ)) to its maximum value (M). The effective number of independent variables can then be calculated as

$$M_{eff}=1+(M-1)\left( 1-\frac{Var\left( \lambda\right)}{M} \right)$$

To obtain the effective number of independent phenotypes from the twelve cortical regions (*M* = 12), we computed the observed variance of the eigenvalues of the correlation matrix of the surface area measures of the twelve cortical regions.

**S8. Twin-based genetic correlation analysis**

In the classical twin study, the observed phenotypic variance **P** in a population can be decomposed into an additive genetic component **A**, a common or shared environmental component C, and a random or unique environmental component **E**,

**P = A + C + E**.

Two representations of the model are equivalent: path coefficients model if variances of all latent variables are standardized, or variance component model otherwise. This is referred to as the ACE model. The twin data enables the estimates of different variance components (21). The monozygotic (MZ) twin pairs correlate 1 for **A**, whereas dizygotic (DZ) pairs correlate 0.5. Both MZ and DZ pairs are assumed to correlate 1 for component **C** (the environmental influences that make twins similar), and uncorrelated for **E** (the environmental influences that make twins differ).

The univariate ACE model can be extended to a bivariate model. The phenotypic covariance matrix can be written as

$$\Sigma_{P}=\Sigma_{A}+\Sigma_{C}+\Sigma_{E}$$

where $\Sigma_{A}$, $\Sigma_{C}$, $\Sigma_{E}$ are $2\times2$ covariance matrices of the additive genetic component, shared environmental component, and random environmental component, respectively. Specifically, the genetic covariance matrix can be written as

$$\Sigma_{A}=\left[ \begin{matrix} \sigma_{A_{11}}^{2} & \sigma_{A_{21}} \\ \sigma_{A_{12}} & \sigma_{A_{22}}^{2} \end{matrix} \right]$$

The genetic correlation *r_g_* is then given by

$$r_{g}=\frac{\sigma_{A_{12}}}{\sigma_{A_{11}}\sigma_{A_{22}}}$$

The variance component estimation for our twin cohort was done with OpenMx, a maximum likelihood based structural equation modeling (SEM) package for genetic data. The analysis of the VETSA twin data using ACE model rendered most estimates of the common environmental variance of surface area measures of cortical regions near zero, and AE models tended to provide the best fit to the data. We therefore dropped the common environmental component in the final analysis and used AE model to infer the variance-covariance matrix of the genetic effects, from which the genetic correlations between pairs of cortical region surface areas were derived.

**S9. Bivariate linear mixed model to estimate genetic correlations using genome-wide SNPs**

A standard bivariate variance component model (22) similar to the bivariate twin model was used to calculate genetic correlations of inter-regional cortical surface areas for the combined-5-cohort. In the model, the phenotype was expressed as a linear function of the sum of additive genetic effects and the residual effects. But different from the twin model, the genetic component, in particular, the genetic relationship matrix, was estimated using the genotype data.

In a standard variance component, or linear mixed, model, a phenotypic trait can be expressed as a linear function of the sum of additive genetic effects and residual effects and written as,

$$y_{j}=\mu+g_{j}+e_{j}$$

where $y_{j}$is the phenotype of individual *j*, *μ* is the overall mean, $e_{j}$ is the residual with $e_{j}\sim N(0, \sigma_{e}^{2})$, and ideally, $g_{j}=\sum_{i=1}^{m} Z_{ji}\mu_{i}$, where *m* is the number of causal loci, $\mu_{i}$ is the scaled additive genetic effect of the *i*th causal variant, and *Z* is the genotypic values standardized across all subjects. However since little is know about causal variants, $g_{j}$ is approximated from genome-wide additive genetic effect of all variants for the individual *j* with $g_{j}\sim N(0, \sigma_{g}^{2})$ (6). The genetic variance $\sigma_{g}^{2}$ captured by all SNPs is estimated by comparing the phenotypic similarity of pairs of distantly related individuals to their SNP-derived genetic similarity, or formally,

$$Var\left( \boldsymbol{Y} \right)= \boldsymbol{V}=\boldsymbol{A}\sigma_{g}^{2}+\boldsymbol{I}\sigma_{e}^{2}$$

where **V** is the variance structure of phenotypic observations, **A** is the genetic relationship matrix, and **I** is an identity matrix. Genetic relationship $A_{jk}$between individuals *j* and *k* is estimated as,

$$A_{jk}=\frac{1}{N}\sum_{i=1}^{N} \frac{(x_{ij}-2p_{i})(x_{ik}-2p_{i})}{2p_{i}(1-p_{i})}$$

where $x_{ij}=0,1,2$ according to whether individual j has genotype bb, Bb or BB at locus *i*, $p_{i}$ is the allele frequency of B allele, and N is the total number of loci. Extending the model to a bivariate linear mixed model (22), the vectors of two phenotypic traits can be then written as

$$\left[ \begin{matrix} y_{1} \\ y_{2} \end{matrix} \right]=\left[ \begin{matrix} 1 & 0 \\ 0 & 1 \end{matrix} \right]\left[ \begin{matrix} \mu_{1} \\ \mu_{2} \end{matrix} \right]+\left[ \begin{matrix} g_{1} \\ g_{2} \end{matrix} \right]+\left[ \begin{matrix} e_{1} \\ e_{2} \end{matrix} \right]$$

with the variance-covariance matrix **V** defined as

$$\boldsymbol{V}=\left[ \begin{matrix} {\boldsymbol{A}\sigma}_{g_{1}}^{2} & \boldsymbol{A}\sigma_{g_{1}g_{2}} \\ {\boldsymbol{A}\sigma}_{g_{1}g_{2}} & \boldsymbol{A}\sigma_{g_{2}}^{2} \end{matrix} \right]+\left[ \begin{matrix} {I\sigma}_{e_{1}}^{2} & I\sigma_{e_{1}e_{2}} \\ {I\sigma}_{e_{1}e_{2}} & I\sigma_{e_{2}}^{2} \end{matrix} \right]$$

where $\sigma_{g_{1}g_{2}}$ is the genetic covariance and $\sigma_{e_{1}e_{2}}$ is the environmental covariance. The genetic correlation between the two traits is defined as

$$r_{g}=\frac{\sigma_{g_{1}g_{2}}}{\sigma_{g_{1}}\sigma_{g_{2}}}$$

The average information restricted maximum likelihood (AIREML) method developed by Lee et al. (23) as implemented in the GCTA package (6) was used to carry out the genetic correlation estimations.

**S10. Mapping brain gene expression data onto the cortical surfaces**

Six adult human brain transcriptomic profiles were obtained from Allen Human Brain Atlas (24, 25). Two individuals had data available on both the left and right hemispheres; the remaining four had data on the left hemisphere only. The individuals were unrelated and composed of three Caucasians, two African Americans, and one Hispanic. There were one female and five males, and aged 42.5 ± 13.4 y (range = [24, 57]). Each individual hemisphere had transcript profiles on 58,692 microarray probes at between 363 and 946 neuroanatomical subdivisions across cerebrum, cerebellum, and brainstem. Based on anatomical locations, we derived a spatial mapping between the 163,842 locations (vertices) in brain imaging analysis, and the cortical subdivisions in transcriptome data, for each of the individual hemispheres.

We first mapped the transcriptome locations to the locations used in the brain imaging analysis. The Allen brain atlas provided data in Montreal Neurological Institute (MNI) coordinates, which was used as the target space for our registration. We registered FreeSurfer space to MNI space by using FreeSurfer’s fsaverage T1 atlas, which was rigidly registered to an MNI T1 brain atlas (colin27). Fig 4 shows the resulting registration between FreeSurfer surface (fsaverage), MNI T1 atlas (colin27 displayed as slice) and Allen brain MNI coordinates displayed as a point cloud. After the registration, gene expression data defined at each point is mapped to FreeSurfer surface vertices by assigning each surface vertex the gene expression of the closest (Euclidean distance) Allen brain atlas coordinate using nearest neighbor interpolation.

Of over 160-million vertices, 2562 vertices on the surface were used to reconstruct the cortical surface areas. Each of the twelve cortical regions was represented by between 168 and 304 vertices, which were mapped to between 14 and 53 transcriptome locations in the cortex.

We used the binary transcriptome data with present/absent flags indicating whether the probe’s expression level was well above the background (which was determined by thresholding on 2-sided t-test p-values (< 0.01) and the difference be- tween signal and background (> 2.6 background standard deviations) (25)). We then collapsed the probe set expression onto the gene level (29180 genes), taking the maximum of the probe sets representing each gene (i.e. if one of the probes representing a gene had value 1, the gene was considered expressed). For each of the cortical regions of an individual hemisphere, whether a gene was expressed was determined by using the majority rule over the locations mapped onto that region from the available transcriptome data. Due to small sample size and near symmetry between the left and right hemispheres with respect to surface area measures of the cortical regions, we aggregated all eight samples (six left hemispheres and two right hemispheres). Whether a gene was expressed in a cortical region was then determined again by using the majority rule over the eight samples, yielding a final consensus set of binary gene expression profiles for the twelve cortical regions for subsequent analysis.

**S11. Gene expression profile similarity based on Jaccard index**

Jaccard index, or Jaccard coefficient, is a measure of similarity between finite sample sets (26). For each cortical region, the gene expression profile can be represented as a gene set: *C_i_* = {*g_k_* | if gene *g_k_* is expressed in cortical region *i*}, where *k* = 1 ... 29180, and *i* = 1 ... 12. The Jaccard coefficient between gene expression profiles of cortical regions *C_i_* and *C_j_* is defended as the fraction of the number of genes expressed in both regions over the total number of genes expressed in at least one of the two regions, or formally,

$$J\left( C_{i}, C_{j} \right)= \frac{\left| C_{i}\cap C_{j} \right|}{\left| C_{i}\cup C_{j} \right|}$$

The Jaccard index has a range of [0,1]. The more similar the gene expression profiles, the larger the Jaccard index between the two profiles.

**S12. Regression analysis with errors in both variables**

There were errors in the estimated genetic correlations between cortical regions using either twin- or genotype-based method. In particular, the *r_g_* estimated by GCTA had large errors in many pairs especially when one or both of the phenotypic traits had low SNP-heritability, i.e., the genetic variance captured by genome-wide SNPs for that trait was low (see S3 Table). For instance, the *r_g_* estimates between precuneus (cluster 11) and any other region was unreliable and essentially noninformative. Ignoring the standard errors therefore would render association or regression analysis much less accurate and informative. In order to realistically compare genetic correlation estimated based on SNPs to that based on twin analysis, we used linear regression with errors-in-both-variables (EIV) model (27, 28). Suppose a linear relationship model of the form $y=\alpha+\beta x$, where both variables have errors,

$$\xi_{i}=x_{i}+\delta_{i}$$

$$\eta_{i}=y_{i}+\epsilon_{i}$$

where $\sigma_{\delta_{i}}^{2}$ and $\sigma_{\epsilon_{i}}^{2}$ are respective variances of $x_{i}$ and $y_{i}$. First, we are interested in establishing a relation between the two measurements. It is not clear which variable should be treated as independent variable and which dependent. Second, with errors in both variables, there is no unique true slope between the two variables (28). We took the approach of total least squares allowing errors in both variables, and minimized the following χ2, assuming that the variable errors were uncorrelated,

$$\sum_{i} \omega_{i}\left( y_{i}-\alpha-\beta x_{i} \right)^{2}$$

where

$$\omega_{i}=\frac{1}{\beta^{2}\sigma_{\delta_{i}}^{2}+\sigma_{\epsilon_{i}}^{2}}$$

We used the strategy described in Numerical Recipes (29) for optimization. The regression of *r_g_* from GCTA on *r_g_* from twin study with errors is shown in S1 Fig. Notice that in the special case where x had no error terms, i.e., $\sigma_{\delta_{i}}=0$, the regression would be equivalent to weighted least square regression. Furthermore, if y had equal variance, i.e., $\sigma_{\epsilon_{i}}^{2}=1$ for standardized y, then the regression would be equivalent to the ordinary least squares.

**S13. Mantel test between correlation or similarity matrices**

Mantel test is a permutation test of the correlation between two distance or similarity matrices (30, 31). Since the mutual independent assumption between elements do not hold for similarly matrices, the significant level of correlation measures cannot be obtained directly from normal probability. Given two symmetrical matrices $X={(x)}_{ij}, Y={(y)}_{ij}, i,j=1\ldots k$, where $x_{ij}$ , $y_{ij}$ represent some similarity or distance measures between elements *i* and *j*, the Mantel coefficient between *X* and *Y* is usually given by

$$r=\frac{\sum_{i<j} \left( x_{ij}-\bar{X} \right)\left( y_{ij}-\bar{Y} \right)}{\left( \sum_{i<j} \left( x_{ij}-\bar{X} \right)^{2}\sum_{i<j} \left( y_{ij}-\bar{Y} \right)^{2} \right)^{1/2}}$$

where $\bar{X}=\frac{1}{n}\sum_{i<j} x_{ij}$,$\bar{Y}=\frac{1}{n}\sum_{i<j} y$, and $n=k(k-1)/2$. Defined as such, the Mantel coefficient is essentially the Pearson’s correlation coefficient between the two vectorized lower triangular parts of the matrices. Since elements within each matrix are distance or similarity measures however, the mutual independence assumption between elements does not hold (variables are not i.i.d.). The significance level of the correlation therefore cannot be derived from normal probability directly. It may instead be obtained by comparing the test coefficient with an empirical null distribution derived from Monte Carlo sampling. We performed 10,000 permutations for each test, rendering the minimum observable significant *p*-value to be 1/10,001. The 95% confidence intervals were obtained with bootstrapping.

The same permutation test was also used in the regression analysis described in the previous section. The matrices were rescaled such that both matrices had the same variance. The slope *β* from the regression with errors-in-both-variables model was reported as the Mantel test coefficient in this analysis and the significant level was determined by the permutation test. When the two correlation matrices did not have explicit error terms, such as when two phenotypic correlation matrices were compared, the coefficient was equivalent to the correlation coefficient as defined in Eq.1. When only one of the matrices had the explicit error terms, for instance between genetic correlation and phenotypic correlation, we regressed the variable with errors onto the one without, which was equivalent to the weighted least square regression. When both matrices had explicit individual error terms, let $\beta_{y\backslash x}$be the slope of regression of *y* on *x*, and $\beta_{x\backslash y}$ of *x* on *y*, then the total least square model allowing errors in both variables warrants that $\beta_{y\backslash x}$ and $\beta_{x\backslash y}$ are reciprocals of each other. This was the case when we compared the genetic relations between twin-based and SNP-based methods. In this case we report the minimum of the two slopes, i.e., $\beta=min(\beta_{y\backslash x}, \beta_{x\backslash y})$, such that *β* is between −1 and 1, to be comparable with correlation coefficients. Specifically, the slope of *r_g_* derived by GCTA regressing on *r_g_* derived by the twin method is reported.

**Consortium Authors**

**The following authors are included under the Pediatric Imaging, Neurocognition and Genetics (PING) Study**

Data used in preparation of this article were obtained from the Pediatric Imaging, Neurocognition and Genetics Study (PING) database ([http://ping.chd.ucsd.edu](http://ping.chd.ucsd.edu/)). Thus, the investigators within PING contributed to the design and implementation of PING and/or provided data but did not participate in analysis or writing of this report. A complete listing of PING investigators can be found at <https://ping-dataportal.ucsd.edu/sharing/Authors10222012.pdf>.

**Coordinating Core:** Terry L. Jernigan (UC San Diego), Connor McCabe, (UC San Diego); **Assessment Core:** Linda Chang, (U Hawaii); Natacha Akshoomoff (UC San Diego), Erik Newman (UC San Diego); **MRI Post-processing Core:** Anders M. Dale (UC San Diego); **MRI Acquisition Core:** Thomas Ernst (U Hawaii), Anders M. Dale (UC San Diego), Peter Van Zijl (KKI), Joshua Kuperman (UC San Diego); **Genetics Core:** Sarah Murray (Scripps Translational Science Institute), Cinnamon Bloss (Scripps Translational Science Institute), Nicholas J. Schork (Scripps Translational Science Institute); **Informatics and Biostatistics:** Mark Appelbaum (UC San Diego), Anthony Gamst (UC San Diego), Wesley Thompson (UC San Diego), Hauke Bartsch (UC San Diego);

**Investigators by Data Collection Site (**FULL PING Investigator Lists):

**University of California, San Diego:** Terry L. Jernigan, Anders M. Dale, Natacha Akshoomoff, **University of Hawaii:** Linda Chang, Thomas Ernst, Brian Keating, **University of California, Davis:** David Amaral, **University of California, Los Angeles:** Elizabeth Sowell, **Kennedy Krieger Institute, Johns Hopkins University:** Walter Kaufmann, Peter Van Zijl, Stewart Mostofsky, **Sackler Institute, Weill Cornell Medical College:** B.J. Casey, Erika J. Ruberry, Alisa Powers, **Massachusetts General Hospital, Harvard University:** Bruce Rosen,, Tal Kenet, **University of Massachusetts:** Jean Frazier, David Kennedy, **Yale University:** Jeffrey Gruen

**PING Methods:**

Data used in the preparation of this article were obtained from the Pediatric Imaging, Neurocognition and Genetics (PING) Study database (<http://ping.chd.ucsd.edu/>). PING was launched in 2009 by the National Institute on Drug Abuse (NIDA) and the Eunice Kennedy Shriver National Institute Of Child Health & Human Development (NICHD) as a 2-year project of the American Recovery and Reinvestment Act. The primary goal of PING has been to create a data resource of highly standardized and carefully curated magnetic resonance imaging (MRI) data, comprehensive genotyping data, and developmental and neuropsychological assessments for a large cohort of developing children aged 3 to 20 years. The scientific aim of the project is, by openly sharing these data, to amplify the power and productivity of investigations of healthy and disordered development in children, and to increase understanding of the origins of variation in neurobehavioral phenotypes. For up-to-date information, see <http://ping.chd.ucsd.edu/>.

**Alzheimer’s Disease Neuroimaging Initiative (ADNI):**

Michael Weiner (UC San Francisco), Paul Aisen (UC San Diego), Ronald Petersen (Mayo Clinic, Rochester), Clifford R. Jack, Jr. (Mayo Clinic, Rochester), William Jagust (UC Berkeley), John Q. Trojanowki (U Pennsylvania), Arthur W. Toga (USC), Laurel Beckett (UC Davis), Robert C. Green (Brigham and Women’s Hospital / Harvard Medical School), Andrew J. Saykin (Indiana University), John Morris (Washington University St. Louis), Leslie M. Shaw (University of Pennsylvania); ADNI External Advisory Board (ESAB): Zaven Khachaturian (Prevent Alzheimer’s Disease 2020), Greg Sorensen (Siemens), Maria Carrillo (Alzheimer’s Association), Lew Kuller (University of Pittsburgh), Marc Raichle (Washington University St. Louis), Steven Paul (Cornell University), Peter Davies (Albert Einstein College of Medicine of Yeshiva University), Howard Fillit (AD Drug Discovery Foundation), Franz Hefti (Acumen Pharmaceuticals), Davie Holtzman (Washington University St. Louis), M. Marcel Mesulman (Northwestern University), William Potter (National Institute of Mental Health), Peter Snyder (Brown University); **ADNI 2 Private Partner Scientific Board (PPSB) Chair:** Adam Schwartz (Eli Lilly); **Data and Publication Committee (DPC)**: Robert C. Green (Brigham and Women’s Hospital/Harvard Medical School (Chair)); **Resource Allocation Review Committee**: Tom Montine (University of Washington (Chair)); **Clinical Core Leaders:** Ronald Petersen (Mayo Clinic, Rochester), Paul Aisen (UC San Diego); **Clinical Informatics and Operations**: Ronald G. Thomas (UC San Diego), Michael Donohue (UC San Diego), Sarah Walter (UC San Diego), Devon Gessert (UC San Diego), Tamie Sather (UC San Diego), Gus Jiminez (UC San Diego); **Biostatistics Core Leaders and Key Personnel:** Laurel Beckett (UC Davis), Danielle Harvey (UC Davis), Michael Donohue (UC San Diego); **MRI Core Leaders and Key Personnel:** Clifford R. Jack, Jr. (Mayo Clinic, Rochester), Matthew Bernstein (Mayo Clinic, Rochester), Nick Fox (University of London), Paul Thompson (Keck School of Medicine of USC), Norbert Schuff (UCSF), Charles DeCarli (UC Davis), Bret Borowski (Mayo Clinic), Jeff Gunter (Mayo Clinic), Matt Senjem (Mayo Clinic), Prashanthi Vemuri (Mayo Clinic), David Jones (Mayo Clinic), Kejal Kantarci (Mayo Clinic), Chad Ward (Mayo Clinic); **PET Core Leaders and Key Personnel:** William Jagust (UC Berkeley)**,** Robert A. Koeppe (University of Michigan), Norm Foster (University of Utah)**,** Eric M. Reiman (Banner Alzheimer’s Institute), Kewei Chen (Banner Alzheimer’s Institute), Chet Mathis (University of Pittsburgh), Susan Landau (UC Berkeley); **Neuropathology Core Leaders:** John Morris (Washington University St. Louis), Nigel J. Cairns (Washington University St. Louis), Erin Householder (Washington University St. Louis), Lisa Taylor-Reinwald (Washington University St. Louis); **Biomarkers Core Leaders and Key Personnel:** J.Q. Trojanowki (UPenn School of Medicine)**,** Les Shaw (UPenn School of Medicine)**,** Virginia M.Y. Lee (UPenn School of Medicine)**,** Magdalena Korecka (UPenn School of Medicine), Michal Figurski (UPenn School of Medicine); **Informatics Core Leaders and Key Personnel:** Arthur W. Toga (USC), Karen Crawford (USC)**,** Scott Neu (USC); **Genetics Core Leaders and Key Personnel:** Andrew J. Saykin (Indiana University), Tatiana M. Foroud (Indiana University)**,** Steven Potkin (UC Irvine), Li Shen (Indiana University), Kelley Faber (Indiana University), Sungeun Kim (Indiana University), Kwangsik Nho (Indiana University)**; Initial Concept Planning & Development:** Michael W. Weiner (UC San Francisco), Leon Thal (UC San Diego), Zaven Khachaturian (Prevent Alzheimer’s Disease 2020); **Early Project Development:** Zaven Khachaturian (Prevent Alzheimer’s Disease 2020), Richard Frank (General Electric), Peter J. Snyder (University of Connecticut), Michael W. Weiner (UC San Francisco), Leon Thal (UC San Diego), Neil Buckholtz (NIA), William Potter (NIMH), Steven Paul (Cornell University), Marilyn Albert (The Johns Hopkins University); **NIA:** John Hsiao (National Institute on Aging/National Institutes of Health) ;

**ADNI Investigators By Site** (FULL ADNI Investigator Lists):

**Oregon Health and Science University:** Jeffrey Kaye, Joseph Quinn, Betty Lind, Raina Carter, Sara Dolen – Past Investigator; **University of Southern California:** Boris A. Gutman, Lon S. Schneider, Sonia Pawluczyk, Mauricio Beccera, Liberty Teodoro, Bryan M. Spann, DO – Past Investigator; **University of California-San Diego:** James Brewer, Helen Vanderswag, Adam Fleisher – Past Investigator; **University of Michigan:** Judith L. Heidebrink, Joanne L. Lord; **Mayo Clinic, Rochester:** Ronald Petersen, Sara S. Mason, Colleen S. Albers, David Knopman, Kris Johnson – Past Investigator; **Baylor College of Medicine:** Rachelle S. Doody, Javier Villanueva-Meyer, Munir Chowdhury, Susan Rountree, Mimi Dang; **Columbia University Medical Center:** Yaakov Stern, Lawrence S. Honig, Karen L. Bell; **Washington University, St. Louis:** Beau Ances, John C. Morris, Maria Carroll, Sue Leon, Erin Householder, Mark A. Mintun – Past Investigator, Stacy Schneider – Past Investigator, Angela Oliver – Past Investigator; **University of Alabama - Birmingham:** Daniel Marson, Randall Griffith, David Clark, David Geldmacher, John Brockington, Erik Roberson; **Mount Sinai School of Medicine:** Hillel Grossman, Effie Mitsis; **Rush University Medical Center:** Leyla deToledo-Morrell, Raj C. Shah; **Wien Center:** Ranjan Duara, Daniel Varon, Maria T. Greig, Peggy Roberts– Past Investigator; **Johns Hopkins University:** Marilyn Albert, Chiadi Onyike, Daniel D’Agostino II, Stephanie Kielb – Past Investigator; **New York University:** James E. Galvin, Dana M. Pogorelec, Brittany Cerbone, Christina A. Michel, Henry Rusinek – Past Investigator, Mony J de Leon – Past Investigator, Lidia Glodzik – Past Investigator, Susan De Santi – Past Investigator; **Duke University Medical Center:** P. Murali Doraiswamy, Jeffrey R. Petrella, Terence Z. Wong; **University of Pennsylvania:** Steven E. Arnold, Jason H. Karlawish, David Wolk; **University of Kentucky:** Charles D. Smith, Greg Jicha, Peter Hardy, Partha Sinha, Elizabeth Oates, Gary Conrad; **University of Pittsburgh:** Oscar L. Lopez, MaryAnn Oakley, Donna M. Simpson; **University of Rochester Medical Center:** Anton P. Porsteinsson, Bonnie S. Goldstein, Kim Martin, Kelly M. Makino – Past Investigator, M. Saleem Ismail – Past Investigator, Connie Brand – Past Investigator; **University of California, Irvine:** Ruth A. Mulnard, Gaby Thai, Catherine Mc-Adams-Ortiz; **University of Texas Southwestern Medical School:** Kyle Womack, Dana Mathews, Mary Quiceno, Ramon Diaz-Arrastia – Past Investigator, Richard King – Past Investigator, Myron Weiner – Past Investigator, Kristen Martin-Cook – Past Investigator, Michael DeVous – Past Investigator; **Emory University:** Allan I. Levey, James J. Lah, Janet S. Cellar; **University of Kansas, Medical Center:** Jeffrey M. Burns, Heather S. Anderson, Russell H. Swerdlow; **University of California, Los Angeles:** Liana Apostolova, Kathleen Tingus, Ellen Woo, Daniel H.S. Silverman, Po H. Lu – Past Investigator, George Bartzokis – Past Investigator; **Mayo Clinic, Jacksonville:** Neill R Graff-Radford, Francine Parfitt, Tracy Kendall, Heather Johnson – Past Investigator; **Indiana University:** Martin R. Farlow, Ann Marie Hake, Brandy R. Matthews, Scott Herring, Cynthia Hunt; **Yale University School of Medicine:** Christopher H. van Dyck, Richard E. Carson, Martha G. MacAvoy; **McGill Univ., Montreal-Jewish General Hospital:** Howard Chertkow, Howard Bergman, Chris Hosein; **Sunnybrook Health Sciences, Ontario:** Sandra Black, Dr Bojana Stefanovic, Curtis Caldwell; **U.B.C. Clinic for AD & Related Disorders:** Ging-Yuek Robin Hsiung, Howard Feldman, Benita Mudge, Michele Assaly, – Past Investigator; **Cognitive Neurology - St. Joseph's, Ontario:** Andrew Kertesz, John Rogers, Dick Trost; **Cleveland Clinic Lou Ruvo Center for Brain Health:** Charles Bernick, Donna Munic; **Northwestern University:** Diana Kerwin, Marek-Marsel Mesulam, Kristine Lipowski, Chuang- Kuo Wu – Past Investigator, Nancy Johnson – Past Investigator; **Premiere Research Inst (Palm Beach Neurology):** Carl Sadowsky, Walter Martinez, Teresa Villena; **Georgetown University Medical Center:** Raymond Scott Turner, Kathleen Johnson, Brigid Reynolds; **Brigham and Women's Hospital:** Reisa A. Sperling, Keith A. Johnson, Gad Marshall, Meghan Frey – Past Investigator; **Stanford University:** Jerome Yesavage, Joy L. Taylor, Barton Lane, Allyson Rosen – Past Investigator, Jared Tinklenberg – Past Investigator; **Banner Sun Health Research Institute:** Marwan N. Sabbagh, Christine M. BeldenSandra A. Jacobson, Sherye A. Sirrel; **Boston University:** Neil Kowall, Ronald Killiany, Andrew E. Budson, Alexander Norbash – Past Investigator, Patricia Lynn Johnson – Past Investigator; **Howard University:** Thomas O. Obisesan, Saba Wolday, Joanne Allard; **Case Western Reserve University:** Alan Lerner, Paula Ogrocki, Leon Hudson – Past Investigator; **University of California, Davis – Sacramento:** Evan Fletcher, Owen Carmichael, John Olichney, Charles DeCarli – Past Investigator; **Neurological Care of CNY:** Smita Kittur; **Parkwood Hospital:** Michael Borrie, T-Y Lee, Dr Rob Bartha; **University of Wisconsin:** Sterling Johnson, Sanjay Asthana, Cynthia M. Carlsson; **University of California, Irvine - BIC:** Steven G. Potkin, Adrian Preda, Dana Nguyen; **Banner Alzheimer's Institute:** Pierre Tariot, Adam Fleisher, Stephanie Reeder; **Dent Neurologic Institute:** Vernice Bates, Horacio Capote, Michelle Rainka; **Ohio State University:** Douglas W. Scharre, Maria Kataki, Anahita Adeli; **Albany Medical College:** Earl A. Zimmerman, Dzintra Celmins, Alice D. Brown; **Hartford Hospital, Olin Neuropsychiatry Research Center:** Godfrey D. Pearlson, Karen Blank, Karen Anderson; **Dartmouth-Hitchcock Medical Center:** Robert B. Santulli, Tamar J. Kitzmiller, Eben S. Schwartz – Past Investigator; **Wake Forest University Health Sciences:** Kaycee M. Sink, Jeff D. Williamson, Pradeep Garg, Franklin Watkins – Past Investigator; **Rhode Island Hospital:** Brian R. Ott, Henry Querfurth, Geoffrey Tremont; **Butler Hospital:** Stephen Salloway, Paul Malloy, Stephen Correia; **UC San Francisco:** Howard J. Rosen, Bruce L. Miller; **Medical University South Carolina:** Jacobo Mintzer, Kenneth Spicer, David Bachman; **St. Joseph’s Health Care:** Elizabether Finger, Stephen Pasternak, Irina Rachinsky, John Rogers, Andrew Kertesz – Past Investigator, Dick Drost – Past Investigator; **Nathan Kline Institute:** Nunzio Pomara, Raymundo Hernando, Antero Sarrael; **University of Iowa College of Medicine:** Susan K. Schultz, Laura L. Boles Ponto, Hyungsub Shim, Karen Elizabeth Smith; **Cornell University:** Norman Relkin, Gloria Chaing, Lisa Raudin; **University of South Florida: USF Health Byrd Alzheimer’s Institute:** Amanda Smith, Kristin Fargher, Balebail Ashok Raj.

**ADNI Methods:**

Data used in the preparation of this article were obtained from the Alzheimer’s Disease Neuroimaging Initiative (ADNI) database (http://adni.loni.usc.edu). The ADNI was launched in 2003 by the National Institute on Aging (NIA), the National Institute of Biomedical Imaging and Bioengineering (NIBIB), the Food and Drug Administration (FDA), private pharmaceutical companies and non-profit organizations, as a $60 million, 5-year public-private partnership. The primary goal of ADNI has been to test whether serial magnetic resonance imaging (MRI), positron emission tomography (PET), other biological markers, and clinical and neuropsychological assessment can be combined to measure the progression of mild cognitive impairment (MCI) and early Alzheimer’s disease (AD). Determination of sensitive and specific markers of very early AD progression is intended to aid researchers and clinicians to develop new treatments and monitor their effectiveness, as well as lessen the time and cost of clinical trials.

The Principal Investigator of this initiative is Michael W. Weiner, MD, VA Medical Center and University of California – San Francisco. ADNI is the result of efforts of many co-investigators from a broad range of academic institutions and private corporations, and subjects have been recruited from over 50 sites across the U.S. and Canada. The initial goal of ADNI was to recruit 800 subjects but ADNI has been followed by ADNI-GO and ADNI-2. To date these three protocols have recruited over 1500 adults, ages 55 to 90, to participate in the research, consisting of cognitively normal older individuals, people with early or late MCI, and people with early AD. The follow up duration of each group is specified in the protocols for ADNI-1, ADNI-2 and ADNI-GO. Subjects originally recruited for ADNI-1 and ADNI-GO had the option to be followed in ADNI-2. For up-to-date information, see http://www.adni-info.org.

**Supplemental References**

1. Kremen WS, Thompson-Brenner H, Leung Y-MJ, Grant MD, Franz CE, Eisen SA, et al. Genes, environment, and time: the Vietnam Era Twin Study of Aging (VETSA). Twin Res Hum Genet. 2006;9(6):1009-22.

2. Espeseth T, Christoforou A, Lundervold AJ, Steen VM, Le Hellard S, Reinvang I. Imaging and cognitive genetics: the Norwegian Cognitive NeuroGenetics sample. Twin Res Hum Genet. 2012;15(3):442-52.

3. Purcell S, Neale B, Todd-Brown K, Thomas L, Ferreira MAR, Bender D, et al. PLINK: a tool set for whole-genome association and population-based linkage analyses. Am J Hum Genet. 2007;81(3):559-75.

4. Pluzhnikov A, Below JE, Konkashbaev A, Tikhomirov A, Kistner-Griffin E, Roe CA, et al. Spoiling the whole bunch: quality control aimed at preserving the integrity of high-throughput genotyping. Am J Hum Genet. 2010;87(1):123-8.

5. Athanasiu L, Mattingsdal M, Kähler AK, Brown A, Gustafsson O, Agartz I, et al. Gene variants associated with schizophrenia in a Norwegian genome-wide study are replicated in a large European cohort. J Psychiatr Res. 2010;44(12):748-53.

6. Yang J, Lee SH, Goddard ME, Visscher PM. GCTA: a tool for genome-wide complex trait analysis. The American Journal of Human Genetics. 2011;88(1):76-82.

7. International HapMap 3 Consortium, Altshuler DM, Gibbs RA, Peltonen L, Altshuler DM, Gibbs RA, et al. Integrating common and rare genetic variation in diverse human populations. Nature. 2010;467(7311):52-8.

8. 1000 Genomes Project Consortium, Abecasis GR, Altshuler D, Auton A, Brooks LD, Durbin RM, et al. A map of human genome variation from population-scale sequencing. Nature. 2010;467(7319):1061-73.

9. Price AL, Patterson NJ, Plenge RM, Weinblatt ME, Shadick NA, Reich D. Principal components analysis corrects for stratification in genome-wide association studies. Nat Genet. 2006;38(8):904-9.

10. Yang J, Benyamin B, McEvoy BP, Gordon S, Henders AK, Nyholt DR, et al. Common SNPs explain a large proportion of the heritability for human height. Nature Genetics. 2010;42(7):565-9.

11. Li Y, Willer CJ, Ding J, Scheet P, Abecasis GR. MaCH: using sequence and genotype data to estimate haplotypes and unobserved genotypes. Genet Epidemiol. 2010;34(8):816-34.

12. Howie B, Fuchsberger C, Stephens M, Marchini J, Abecasis GR. Fast and accurate genotype imputation in genome-wide association studies through pre-phasing. Nat Genet. 2012;44(8):955-9.

13. Siva N. 1000 Genomes project. Nat Biotechnol. 2008;26(3):256.

14. Dale AM, Fischl B, Sereno MI. Cortical surface-based analysis. I. Segmentation and surface reconstruction. Neuroimage. 1999;9(2):179-94.

15. Fischl B, Sereno MI, Dale AM. Cortical surface-based analysis. II: Inflation, flattening, and a surface-based coordinate system. Neuroimage. 1999;9(2):195-207.

16. Chen C-H, Panizzon MS, Eyler LT, Jernigan TL, Thompson W, Fennema-Notestine C, et al. Genetic influences on cortical regionalization in the human brain. Neuron. 2011;72(4):537-44.

17. Chen C-H, Gutierrez ED, Thompson W, Panizzon MS, Jernigan TL, Eyler LT, et al. Hierarchical genetic organization of human cortical surface area. Science. 2012;335(6076):1634-6.

18. Wood SN. Generalized additive models : an introduction with R. Texts in statistical science Boca Raton: Chapman \& Hall/CRC. 2006;xvii:392.

19. Cheverud JM. A simple correction for multiple comparisons in interval mapping genome scans. Heredity (Edinb). 2001;87(Pt 1):52-8.

20. Nyholt DR. A simple correction for multiple testing for single-nucleotide polymorphisms in linkage disequilibrium with each other. The American Journal of Human Genetics. 2004;74(4):765-9.

21. Neale M, Cardon L. Methodology for Genetic Studies of Twins and Families. 6th ed: Springer; 1992.

22. Thompson R. The Estimation of Variance and Covariance Components with an Application when Records are Subject to Cullingan application when records are subject to culling. Biometrics. 1973;29(3):527-50.

23. Lee SH, Yang J, Goddard ME, Visscher PM, Wray NR. Estimation of pleiotropy between complex diseases using single-nucleotide polymorphism-derived genomic relationships and restricted maximum likelihood. Bioinformatics. 2012;28(19):2540-2.

24. Hawrylycz MJ, Lein ES, Guillozet-Bongaarts AL, Shen EH, Ng L, Miller JA, et al. An anatomically comprehensive atlas of the adult human brain transcriptome. Nature. 2012;489(7416):391-9.

25. Allen Institute for Brain Science. Allen Human Brain Atlas [Internet]. Available from: <http://human.brain-map.org/>. 2014.

26. Jaccard P. Étude comparative de la distribution florale dans une portion des Alpes et des Jura. Bulletin de la Société Vaudoise des Sciences Naturelles. 1901;37:547--79.

27. Murtagh F. Linear regresssion with errors in both variables: A short review. Bias and Uncertainties in Astronomy. 1990.

28. Akritas MG, Bershady MA. Linear Regression for Astronomical Data with Measurement Errors and Intrinsic Scatter. The Astrophysical Journal. 1996;470:706-14.

29. Press WH, Teukolsky SA, Vetterling WT, Flannery BP. Numerical Recipes in Fortran 77. The Art of Scientific Computing. 2nd ed: Cambridge University Press; 1992 September.

30. Mantel N. The detection of disease clustering and a generalized regression approach. Cancer Res. 1967;27(2):209-20.

31. Smouse PE, Long JC, Sokal RR. Multiple Regression and Correlation Extensions of the Mantel Test of Matrix Correspondence. Systematic Zoology. 1986;35(4):627-32.
